# Crotonylated BEX2 interacts with NDP52 and enhances mitophagy to modulate chemotherapeutic agent-induced apoptosis in non-small-cell lung cancer cells

**DOI:** 10.1038/s41419-023-06164-6

**Published:** 2023-09-30

**Authors:** Ning Mu, Yu Wang, Xiaopeng Li, Zhiyuan Du, Yingdi Wu, Min Su, Yingying Wang, Xiaoyang Sun, Ling Su, Xiangguo Liu

**Affiliations:** 1 Shandong Provincial Key Laboratory of Animal Cell and Developmental Biology, School of Life Sciences, Shandong University, Qingdao, China; 2 The Second Hospital, Shandong University, Jinan, China; 3 Qilu Hospital, Shandong University, Jinan, China

**Keywords:** Non-small-cell lung cancer, Mitophagy, Apoptosis

## Abstract

Brain expressed X-linked gene 2 (*BEX2*) encoded protein was originally identified to promote transcription by interacting with several transcription factors in the DNA–binding complexes. Recently, BEX2 was found to be localized in cytosol and/or mitochondria and regulate apoptosis in cancer cells and tumor growth. However, the molecular mechanism underlying its roles in cancer cells remains unclear. Here, we report that crotonylated BEX2 plays an important role in inhibiting chemotherapeutic agent-induced apoptosis via enhancing mitophagy in human lung cancer cells. BEX2 promotes mitophagy by facilitating interaction between NDP52 and LC3B. Moreover, BEX2 crotonylation at K59 is critical in the BEX2-mediated mitophagy in lung cancer cells. The K59R mutation of BEX2 inhibits mitophagy by affecting the interaction of NDP52 and LC3B. BEX2 expression is elevated after anticancer drug treatment, and its overexpression inhibits chemotherapy-induced apoptosis. In addition, inhibition of BEX2-regulated mitophagy sensitizes tumor cells to apoptosis. Furthermore, BEX2 promotes tumor growth and inhibits apoptosis by regulating mitophagy in vivo. We also confirm that BEX2 is overexpressed in lung adenocarcinoma and is associated with poor prognosis in lymph node metastasis-free cancer. Therefore, combination treatment with pharmaceutical approaches targeting BEX2-induced mitophagy and anticancer drugs may represent a potential strategy for NSCLC therapy.

## Introduction

*BEX2* gene belongs to the brain expressed X-linked gene family (brain expressed X-linked gene 2). It encodes four isoforms of BEX2 protein, and the longest one is composed of 160 amino acid residues [1, 2]. It was reported that BEX2 interacted with the transcription factors LMO2, NSCL2, or LDB1 in a DNA-binding complex and modulated transcription [3]. Recent studies have revealed that BEX2 also localized in cytosol and/or mitochondria [2, 4]and regulated apoptosis in cancer cells and tumor growth. BEX2 overexpression promotes cell growth and survival in breast cancer cells, indicating its pro-oncogenic function in this disease [5]. BEX2 was highly expressed and required for maintaining dormant cancer stem cells in human cholangiocarcinoma and hepatocellular carcinoma (HCC) [6]. Resistance to chemotherapeutic agents, such as cisplatin and gemcitabine in patients was associated with the upregulation of BEX2 in different cancer types, like HCC and pancreatic adenocarcinoma [6, 7]. How BEX2 regulates chemoresistance in cancer cells is yet to be identified.

Mitochondria, the biosynthetic, bioenergetic, and signaling organelles, are crucial for physiological adaptations and cellular stress responses to the environment [8–10]. The damaged or dysfunctional mitochondria are related to aging, cancer, and many other diseases [11–13]. Therefore their proper removal is necessary for organismal health [14]. Mitophagy is a mitochondrial quality control mechanism that selectively removes damaged mitochondria via macroautophagy [15], which is mediated by PINK1-Parkin [16, 17] or several other ubiquitin E3 ligases such as MUL1, SIAH1, ARIH1, and TRAF2 [18–21]. Emerging evidence indicates that mitophagy is involved in chemoresistance. In breast cancer, it has been reported that the autophagy/mitophagy inhibitor liensinine potently enhances the efficacy of apoptosis [22]. An increasing number of factors have been found to inhibit apoptosis in cancer cells in a mitophagy-dependent manner.

NDP52 belongs to a group of autophagy receptors which includes OPTN, p62, TAX1BP1, and NBR1, etc. They are adaptors that can bind both ubiquitinated protein and LC3 to induce mitophagy [18, 23, 24]. BEX2 could be an interactor of NDP52 identified by the yeast two-hybrid [25]. The biological significance of this interaction is still unclear. In addition, the activity and stability of BEX2 are controlled by post-translational modifications such as ubiquitination [2]. Crotonylation was initially reported on the lysine residues of histones enriched in promoter and enhancer regions [26]. Subsequently, non-histone crotonylation was identified [27], and crotonylation was reported to share multiple current enzymes with acetylation, such as p300, PCAF, MOF, HDAC1/2/3, and SIRT1/2/3 [28–30]. Crotonylated proteins play an important role in the regulation of multiple biological processes, such as spermatogenesis, gene expression and the cell cycle [31–33]. Acox2 is a regulator of non-histone lysine crotonylation that might play a critical role in hepatic metabolic homeostasis [34]. However, the functional elucidation of non-histone crotonylation is still in its infancy.

Here, we investigated how BEX2 regulated cancer cell apoptosis in a mitophagy-dependent manner. We showed that BEX2 regulated mitophagy by promoting the interaction of NDP52 and LC3B. Moreover, we found that crotonylation of BEX2 at K59 was essential for its function. And we verified that BEX2 inhibited apoptosis by elevating mitophagy. Clinically, the level of BEX2 is upregulated in LUAD and is associated with poor prognosis in lymph node metastasis-free cancer. Our data suggest that targeting BEX2 might be an attractive therapeutic strategy for lung cancer treatment.

## Results

### BEX2 promotes mitophagy in NSCLC cells

**Fig. 1:**
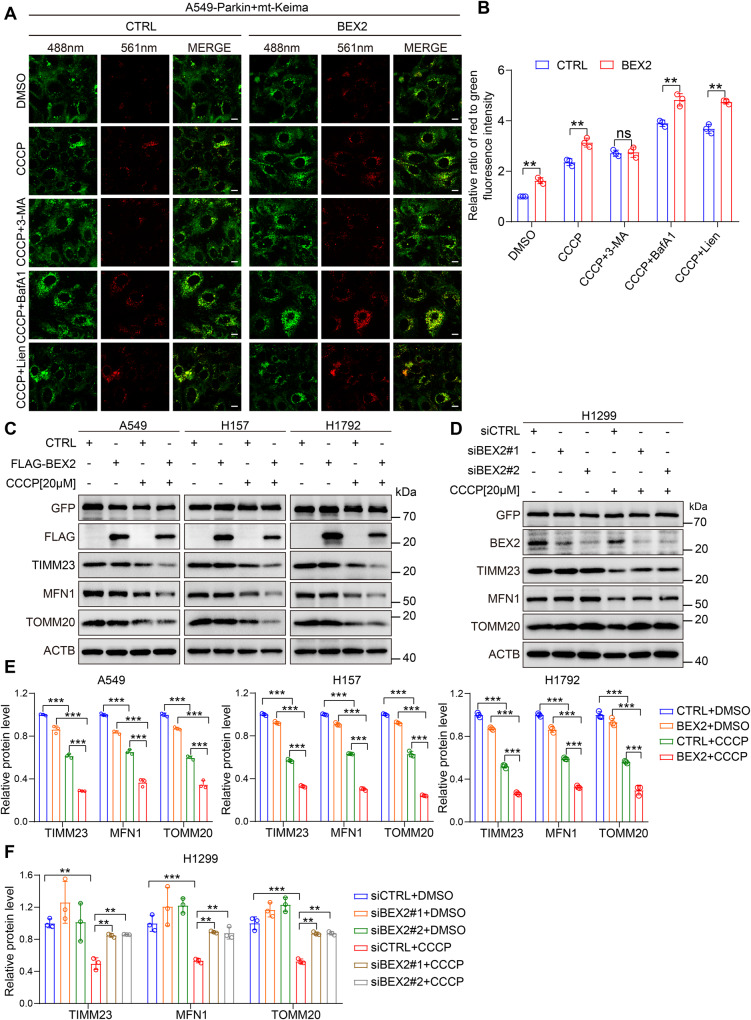
BEX2 promotes mitophagy in NSCLC cells. **A**, **B** A549 cells were co-transfected with HA-Parkin and mt-Keima, and then the cells were transfected with CTRL(pcDNA3.1) or BEX2. Cells were pre-treated with BafilomycinA1(20 nM), liensinine (20 μM), and 3-MA (5 mM) for 0.5 h then treated with CCCP (20 μM) for 6 h. Mitophagy flux was monitored by confocal microscopy. Green indicates mito-Keima fluorescence excited at 488 nm (measuring mitochondria with a neutral pH), red indicates mt-Keima fluorescence excited at 561 nm (measuring mitochondria with an acidic pH) (**A**). Quantification of the relative ratio of fluorescence intensity (561 nm: 488 nm) of the cells (**B**). Data are presented as the mean ± SD (*n* = 3 independent experiments, 20 cells per experiment), and statistical significance was assessed by two-way ANOVA. **P < 0.01, ns not significant. Scale bars: 10 μm. **C** A549, H157, and H1792 cells were transfected with GFP-Parkin and CTRL(pcDNA3.1) or BEX2, then cells were further incubated with CCCP (20 μM) for 6 h. Cell lysates were analyzed by western blotting with the indicated antibodies. **D** H1299 cells were transfected with GFP-Parkin and siCTRL or siBEX2#1, siBEX2#2, and then cells were further incubated with CCCP (20 μM) for 6 h. Cell lysates were analyzed by western blotting with the indicated antibodies. **E** The relative protein levels in A549, H157, and H1792 cells (**C**) were further evaluated by densitometry analysis using ImageJ software and quantified for the ratio of TIMM23/MFN1/TOMM20: ACTB. Data are presented as the mean ± SD (*n* = 3 independent experiments), and statistical significance was assessed by one-way ANOVA. ***P < 0.001. **F** The relative protein levels in H1299 cells (**D**) were further evaluated by densitometry analysis using ImageJ software and quantified for the ratio of TIMM23/MFN1/TOMM20: ACTB. Data are presented as the mean ± SD (*n* = 3 independent experiments), and statistical significance was assessed by one-way ANOVA. **P < 0.01, ***P < 0.001.

BEX2 was shown to be localized in both the cytosolic and the mitochondrial fractions (Supplementary Fig. 1B). CCCP, a mitophagy inducer, elevated the mitochondrial accumulation of BEX2 (Supplementary Fig. 1C). BEX2 overexpression promoted CCCP-induced mitophagy, as assessed by the increased red to green ratio of mt-Keima (Fig. 1A and B). and the reduced level of several mitochondrial markers, such as TIMM23, MFN1, and TOMM20 (Fig. 1C and E) in A549-Parkin, H157-Parkin, and H1792-Parkin cells. Consistently, BEX2 ablation by its siRNA reduced the red fluorescence intensity of mt-Keima upon CCCP treatment and prevented mitochondrial protein degradation while CCCP inducing mitophagy in H1299-Parkin cells (Fig. 1D, F and Supplementary Fig. 1D, E). As shown in Fig. 1A, overexpression of BEX2 further increased the red to green ratio of mt-Keima under the autophagy inhibitors BafilomycinA1(BafA1) and liensinine (Lien) treatment. While for initiating autophagy inhibitor 3-MA treatment, the red to green ratio of mt-Keima did not vary significantly in the BEX2 overexpressed group compared with the control group. Besides, the treatment with autophagy inhibitors prevented the reduction of TIMM23, MFN1 and TOMM20 induced by overexpression of BEX2 (Supplementary Fig. 1F-K). These results showed that BEX2 could act in the initiation of mitophagy and promote the mitophgic flux.

Because the endogenous Parkin in most lung cancer cell lines is expressed minimally [35], we next evaluated the role of BEX2-regulated mitophagy in lung cancer cell lines without overexpression of Parkin. Interestingly, BEX2 still dramatically reduced levels of mitochondrial proteins under CCCP or valinomycin treatment (Supplementary Fig. 2A, B), indicating that BEX2 also promoted mitophagy independently of Parkin. CCCP-induced mitophagy was then measured in BEX2-depleted cells. Depletion of BEX2 alleviated the reduction of TOMM20 and TIMM23, which were reversed by re-expression of BEX2 (Supplementary Fig. 1C, D), further confirming that BEX2 is involved in the decrease of mitochondrial proteins upon mitophagy. We propose that BEX2 promotes mitophagy dependently or independently of the PINK1-Parkin pathway.

### BEX2 interacts with NDP52 and LC3B to regulate mitophagy in NSCLC cells

**Fig. 2:**
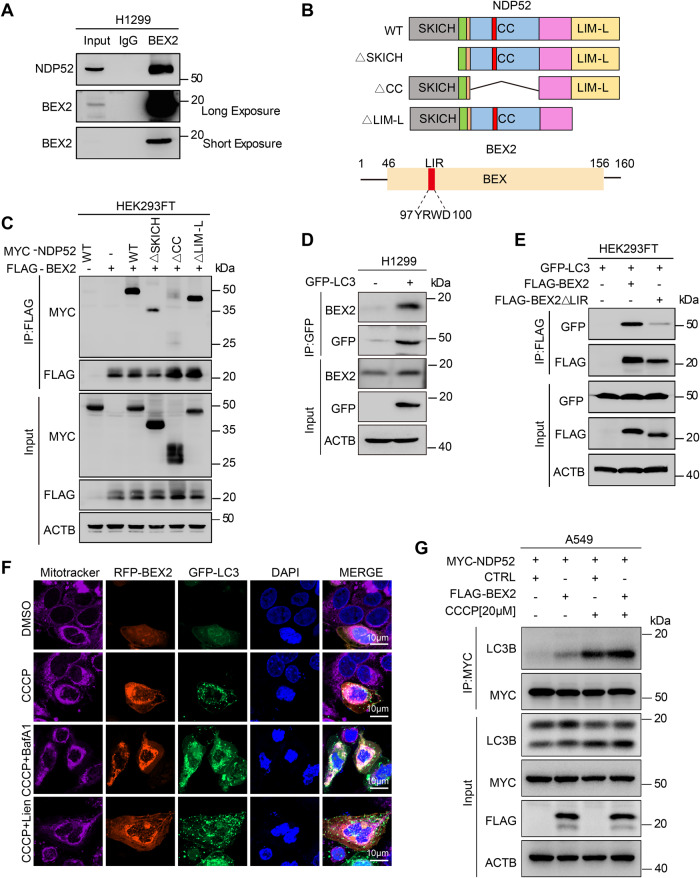
BEX2 interacts with NDP52 and LC3B to regulate mitophagy in NSCLC cells. **A** Co-IP assays were carried out with the BEX2 antibody or IgG in H1299 cells followed by western blotting using anti-BEX2 and anti-NDP52 antibodies. **B** Schematic diagram of NDP52 and the domain-deleted constructs (top). Schematic diagram of BEX2; LIR, LC3-interacting region (bottom). **C** HEK293FT cells were co-transfected with MYC-NDP52 or MYC-tagged domain-deleted mutants of NDP52 together with CTRL (pcDNA3.1) or FLAG-BEX2, and then co-IP assays were carried out with an MYC antibody followed by western blotting using the indicated antibodies. **D** H1299 cells were transfected with CTRL (pcDNA3.1) or GFP-LC3, and then co-IP assays were carried out with GFP antibody followed by western blotting using anti-GFP and anti-BEX2 antibodies. **E** HEK293FT cells were transfected with FLAG-BEX2 or FLAG-tagged LIR-deleted mutants of BEX2 together with GFP-LC3B, and then co-IP assays were carried out with FLAG antibody followed by western blotting using anti-BEX2 and anti-GFP antibodies. **F** A549 cells were transfected with RFP-BEX2 and GFP-LC3, and then cells were pre-treated with BafilomycinA1(20 nM) and Liensinine (20 μM) for 0.5 h then incubated with CCCP (20 μM) for 6 h. MitoTracker Deep Red labeled mitochondria. The co-localization with BEX2, LC3 and mitochondria was monitored by confocal microscopy. **G** A549 cells were co-transfected with MYC-NDP52 and CTRL(pcDNA3.1) or BEX2, and then the cells were incubated with CCCP (20 μM) for 6 h. Cell lysates were incubated with an MYC antibody by co-IP assays followed by western blotting using the indicated antibodies.

To verify the interaction between BEX2 and NDP52, the co-immunoprecipitations (co-IPs) were performed. As shown in Fig. 2A, Supplementary Fig. 3A, B, NDP52 binds to BEX2 specifically. Human NDP52 consists of an amino-terminal skeletal muscle and kidney-enriched inositol phosphatase carboxyl homology (SKICH) domain (AA, 1–127), an intermediate coiled-coil (CC) domain (AA, 134–350) and a carboxy-terminal LIM-like (LIM-L) domain (AA, 395–446) [36]. FLAG-BEX2 and full length or truncated NDP52 with different deletions (Fig. 2B) were co-transfected into HEK293FT cells. Co-IPs demonstrated that the deletion of the CC domain but not the SKICH or LIM-L domain blocked the interaction between NDP52 and BEX2, indicating that the CC domain of NDP52 might be critical for its interaction with BEX2 (Fig. 2C).

As an autophagy receptor, NDP52 is known to interact with LC3B via its LIR motif and then link cargo to the autophagosomal membrane. The co-IP demonstrated that BEX2 also interacted with LC3B (Fig. 2D). Deep analysis revealed a potential LIR motif (YRWD, aa97-100) in the BEX2 protein sequence (Fig. 2B). The YRWD deletion or mutation (Y97A/W99A) of BEX2 both attenuated the binding between BEX2 and LC3B (Fig. 2E and Supplementary Fig. 3C), suggesting its importance in BEX2-LC3B interaction. We also detected the co-localization with BEX2 and LC3B in mitophagy. We co-transfected with RFP-BEX2 and GFP-LC3B in A549 cells, and then cells were treated with CCCP, BafilomycinA1, and liensinine. We used MitoTracker Deep Red labeled mitochondria. Besides, we have examined the co-localization with BEX2, LC3B and mitochondria in mitophagy under CCCP, BafilomycinA1 and liensinine treatment by confocal microscopy. The data showed that BEX2 co-located with LC3B on the mitochondria in mitophagy, and BEX2 co-located more with LC3B under BafilomycinA1 and liensinine treatment (Fig. 2F). Then we investigated the effect of BEX2 on the NDP52-mediated mitophagy induced by CCCP. The co-IP showed that NDP52-LC3B interaction was enhanced by BEX2 overexpression in A549 cells (Fig. 2G) and impaired while BEX2 was knocked down (Supplementary Fig. 3D). The NDP52 knockdown weakened BEX2-promoted mitophagy upon CCCP treatment in A549-Parkin cells (Supplementary Fig. 3E). Thus, it can be concluded that BEX2 promotes mitophagy by enhancing the NDP52-LC3B interaction.

### Crotonylation modification of BEX2 is required for mitophagy regulation

**Fig. 3:**
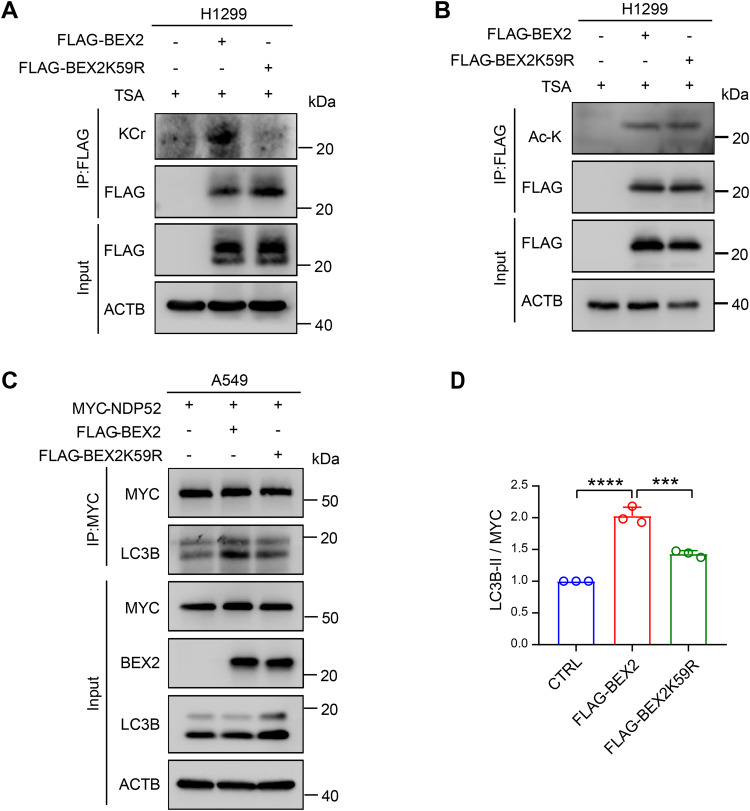
Crotonylation modification of BEX2 is required for mitophagy regulation. **A** IP analyses for FLAG and crotonylation of BEX2 in H1299 cells expressing CTRL(pcDNA3.1), FLAG-BEX2 or FLAG-BEX2 K59R with TSA (3 μM, 12 h) treatment. **B** IP analyses for FLAG and acetylation of BEX2 in H1299 cells expressing CTRL(pcDNA3.1), FLAG-BEX2 or FLAG-BEX2 K59R with TSA (3 μM, 12 h) treatment. **C**, **D** A549 cells were co-transfected with MYC-NDP52 and BEX2 or BEX2K59R, and then the cells were incubated with CCCP (20 μM) for 6 h. Cell lysates were incubated with MYC antibody by co-IP assays followed by western blotting using the indicated antibodies (**C**). The relative protein levels were further evaluated by densitometry analysis using ImageJ software and quantified for the ratio of LC3B-II: MYC in IP (**D**). Data are presented as the mean ± SD (*n* = 3 independent experiments), and statistical significance was assessed by one-way ANOVA. ***P < 0.001, ****P < 0.0001.

Post-translational modifications are a common property for many proteins that can regulate autophagy [37]. Flag-tagged human BEX2 protein was pulled down and subjected to tandem mass spectrometry (MS/MS). The crotonylation modification at lysine 59 (K59) was identified in BEX2 (Supplementary Fig. 4A). The crotonylation of BEX2 was validated by western blot with a specific antibody against crotonylated lysine following the BEX2 pulldown. Furthermore, the replacement of K59 with arginine (K59R) dramatically reduced the crotonylation level of BEX2, indicating that K59 is convincingly the crotonylation site of BEX2 (Fig. 3A). In comparison, K59R did not affect the acetylation of BEX2 (Fig. 3B), emphasizing that the K59 of BEX2 is not an acetylation site but a crotonylation site. To characterize the biological impact of the BEX2 crotonylation, firstly, we performed a co-IP assay to examine its effect on the interaction of NDP52 with LC3B, showing that BEX2(K59R) failed to facilitate this interaction (Fig. 3C, D). Then CCCP or valinomycin was applied to induce mitophagy in BEX2 or BEX2(K59R) overexpressed A549-Parkin cells. The BEX2(K59R) overexpression showed minor enhancement of mitophagy (Supplementary Fig. 4B). These data suggest that crotonylation of BEX2 is indispensable for interacting NDP52 with LC3B and regulating mitophagy.

### BEX2 inhibits chemotherapeutic agent-induced apoptosis

**Fig. 4:**
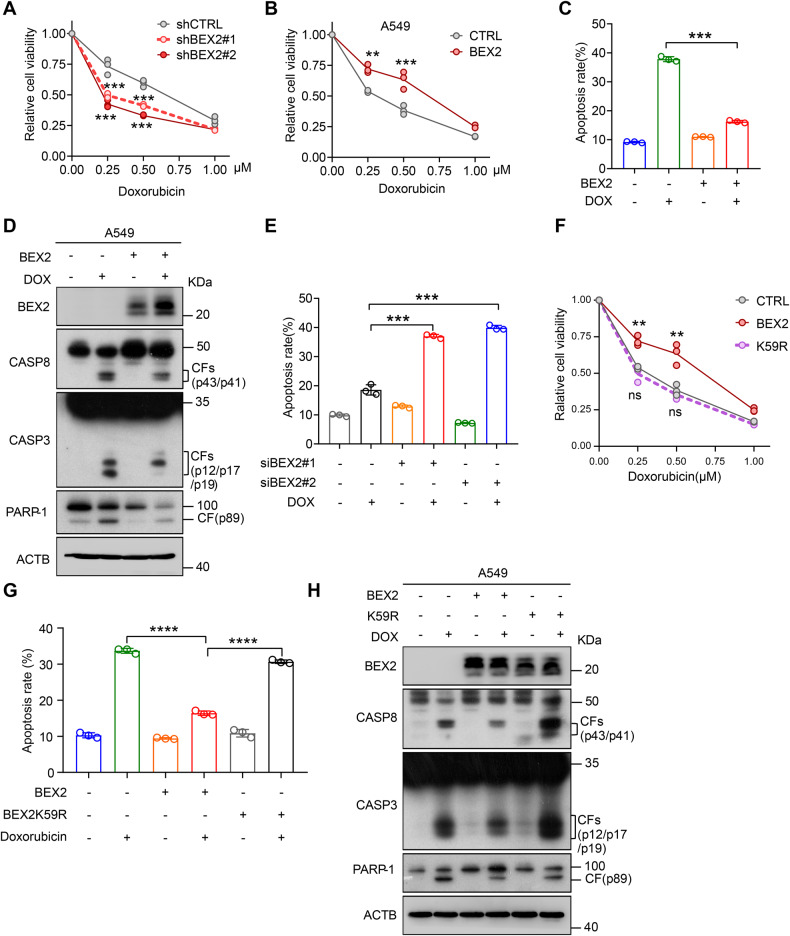
BEX2 inhibits chemotherapeutic agent-induced apoptosis. **A** CCK-8 assays were performed in stably transduced H1299 cells, which were treated with doxorubicin (0, 0.25, 0.5, 1 μM) for 24 h. Data are presented as the mean ± SD (*n* = 3), and statistical significance was assessed by two-way ANOVA. ***P < 0.001. **B** CCK-8 assays were performed in stably transduced A549 cells which were treated with doxorubicin (0, 0.25, 0.5, 1 μM) for 24 h. Data are presented as the mean ± SD (*n* = 3), and statistical significance was assessed by two-way ANOVA. **P < 0.01, ***P < 0.001. **C** A549 cells were transfected with CTRL(pcDNA3.1) or BEX2 and then treated with doxorubicin for 24 h, cells were stained by Annexin V-FITC/7-AAD and flow cytometry analysis. Data are presented as the mean ± SD (*n* = 3), and statistical significance was assessed by two-tailed Student’s *t*-test. ***P < 0.001. **D** A549 cells were transfected with CTRL(pcDNA3.1) or BEX2 and then incubated with doxorubicin (DOX, 1 μM) for 24 h. Cell lysates were analyzed by western blotting with the indicated antibodies. **E** H1299 cells were transfected with siCTRL or siBEX2 and then treated with doxorubicin for 24 h, cells were stained by Annexin V-FITC/7-AAD and flow cytometry analysis. Data are presented as the mean ± SD (*n* = 3), and statistical significance was assessed by two-tailed Student’s *t*-test. ***P < 0.001. **F** CCK-8 assays were performed in stably transduced A549 cells, which were treated with doxorubicin (0, 0.25, 0.5, 1 μM) for 24 h. Data are presented as the mean ± SD (*n* = 3), and statistical significance was assessed by two-way ANOVA. **P < 0.01, ns, not significant for the indicated comparison. **G** A549 cells were transfected with CTRL(pcDNA3.1), BEX2 and BEX2K59R. After treatment with doxorubicin for 24 h, cells were stained with Annexin V-FITC/7-AAD and detected by flow cytometry analysis. Data are presented as the mean ± SD (*n* = 3), and statistical significance was assessed by two-tailed Student’s *t*-test. ****P < 0.0001. **H** A549 cells were transfected with CTRL(pcDNA3.1), BEX2 or BEX2K59R, and then incubated with doxorubicin (DOX, 1 μM) for 24 h. Cell lysates were analyzed by western blotting with the indicated antibodies.

Increasing evidence indicates that inhibiting mitophagy could reinforce the efficacy of chemotherapy by enhancing cancer cell death [38, 39]. We hypothesized that BEX2 might promote mitophagy to resist cell apoptosis in cancer cells. To investigate the potential role of BEX2 on apoptosis in NSCLC cells. We first treated H1299 cells with several clinically available anticancer drugs (e.g., doxorubicin (DOX), pemetrexed (PEM) and cisplatin). Interestingly, doxorubicin, pemetrexed and cisplatin markedly increased the expression of BEX2 with apoptosis induction (Supplementary Fig. 5A–C), suggesting that BEX2 might be involved in the regulation of anticancer drugs-induced apoptosis. Therefore, we next investigated whether modulation of BEX2 expression had an impact on cell survival. To accomplish this, H1299 cells stably knocking down endogenous BEX2 were treated with anticancer drugs, and we observed that cell viability was significantly decreased in BEX2-knockdown cells after doxorubicin or cisplatin treatment compared with control cells (Fig. 4A and Supplementary Fig. 5D, E), suggesting a protective effect of BEX2 following doxorubicin treatment.

In contrast, overexpression of BEX2 was sufficient to increase the ability of A549 cells to survive after anticancer drug treatment (Fig. 4B and Supplementary Fig. 5F). Indeed, overexpression of BEX2 markedly decreased apoptotic signaling induced by doxorubicin and pemetrexed. Similar results were obtained by flow cytometry (Fig. 4C, D and Supplementary Fig. 5G, H). Then, we used Hoechst 33342 staining for apoptosis analysis. Overexpression of BEX2 suppressed the apoptosis induced by doxorubicin (Supplementary Fig. 5I). To further investigate BEX2-inhibited apoptosis in NSCLC cells, two independent siRNAs targeting BEX2 sensitized H1299 cells to doxorubicin-induced apoptosis, as shown by an increase in CASP3 and PARP-1 cleavage (Supplementary Fig. 6A). Moreover, compared with the control group, the knockdown of BEX2 induced an increase in the percentage of apoptosis by Annexin V-FITC/7-AAD staining and flow cytometry analysis (Fig. 4E and Supplementary Fig. 6B). This sensitization to doxorubicin-induced apoptosis following BEX2 knockdown was confirmed by Hoechst 33342 staining analysis (Supplementary Fig. 6C). Collectively, these data indicate that BEX2 might inhibit chemotherapeutic agent-induced apoptosis in certain NSCLC cells.

We then explored the effect of BEX2 crotonylation on the chemotherapeutic agent-induced apoptosis in cancer cells. BEX2 increased cell viability upon doxorubicin treatment, while BEX2K59R abolished this effect (Fig. 4F and Supplementary Fig. 6D). Moreover, BEX2K59R failed to alleviate the apoptosis induced by doxorubicin. The flow cytometry analysis also showed that overexpression of BEX2K59R increased the levels of apoptosis compared with the group of BEX2 overexpression (Fig. 4G, H and Supplementary Fig. 6E), suggesting that BEX2 crotonylation modification plays a critical role in inhibiting apoptosis in NSCLC cancer cells.

### Inhibition of BEX2-regulated mitophagy sensitizes tumor cells to chemotherapy-induced apoptosis

**Fig. 5:**
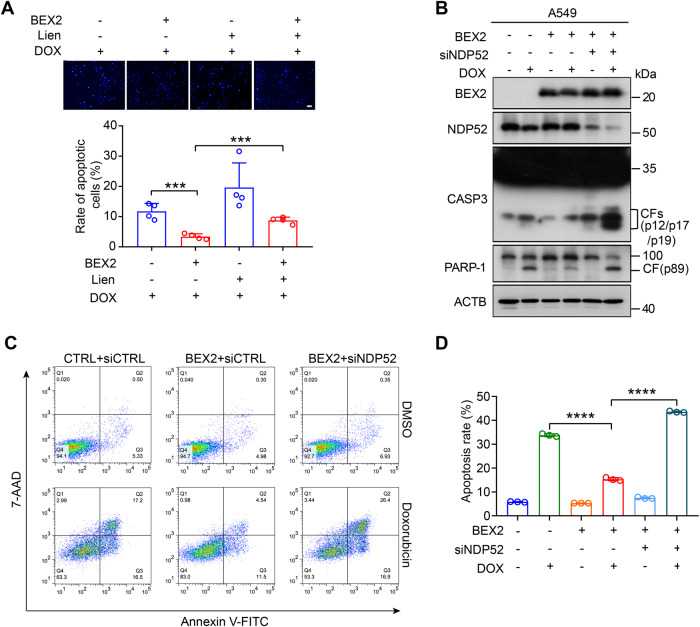
Inhibition of BEX2-regulated mitophagy sensitizes tumor cells to chemotherapy-induced apoptosis. **A** A549 cells stably overexpressing BEX2 were pretreated with liensinine (Lien, 20 μM) for 0.5 h and then combined with doxorubicin for the next 24 h. Hoechst 33342 staining analysis of cell apoptosis. Data are presented as the mean ± SD (*n* = 3 independent experiments, 20 cells per experiment), and statistical significance was assessed by two-tailed Student’s *t*-test. ***P < 0.001. Scale bar: 50 μm. **B**–**D** A549 cells were transfected with BEX2 and NDP52 siRNA, and then treated with doxorubicin for 24 h. Cell lysates were analyzed by western blotting with the indicated antibodies (**B**). Cells were stained with Annexin V-FITC/7-AAD and detected by flow cytometry analysis (**C**, **D**). Data are presented as the mean ± SD (*n* = 3), and statistical significance was assessed by two-tailed Student’s *t*-test. ****P < 0.0001.

Consistent with previous reports, our data revealed that liensinine (Lien), an inhibitor of mitophagy that blocks autophagosome-lysosome fusion [22], aggravated doxorubicin-induced apoptosis (Supplementary Fig. 7A, B). To further evaluate whether BEX2 inhibited apoptosis in NSCLC cells through the modulation of mitophagy, we treated with liensinine to inhibit mitophagy in BEX2-overexpressed cells. We found that the BEX2 overexpression-induced decrease in apoptosis was impaired by liensinine (Fig. 5A). We have proved that BEX2 interacted with NDP52 to promote mitophagy. To explore whether BEX2 inhibited apoptosis via NDP52-specific mitophagy, we knocked down NDP52 expression by siRNA and then measured the cleavage of CASP3 and PARP-1 in BEX2-overexpressed cells. The cleavage of CASP3 and PARP-1 were augmented in NDP52 depleted and BEX2 overexpressed cells compared with the group of only BEX2 overexpression (Fig. 5B). Furthermore, we found that the knockdown of NDP52 reversed BEX2-induced decrease of apoptosis rate using Annexin V-FITC/7-AAD staining and flow cytometry analysis (Fig. 5C, D). Together, our data show that BEX2 inhibits apoptosis via activation of NDP52-dependent mitophagy. Moreover, we also found that the knockdown of endogenous BEX2 was sufficient to reduce the ability of cells to survive and increase apoptosis after CCCP treatment, implying the protective role of BEX2 against mitochondrial damage (Supplementary Fig. 7C–E).

We also transfected A549 cells with siRNAs to block the expression of ATG5 or ATG7. We then measured the expression of apoptosis-related proteins after treatment with BEX2 overexpression. Interestingly, in BEX2-overexpressed cells, knockdown of ATG5 or ATG7 led to increased cleavage of CASP8, CASP3, and PARP-1 compared with control cells (Supplementary Fig. 8A, B). we also used siRNAs to knock down the expression of ATG5 or ATG7 in BEX2-overexpressed cells and measured the percentage of apoptosis by flow cytometry. The results showed that the knockdown of ATG5 or ATG7 reversed the decrease of apoptosis rate by BEX2 overexpression (Supplementary Fig. 8C–F). Taken together, these results demonstrate that BEX2 inhibits apoptosis via activation of the mitophagy pathway.

### BEX2-regulated mitophagy promotes tumor growth in vivo

**Fig. 6:**
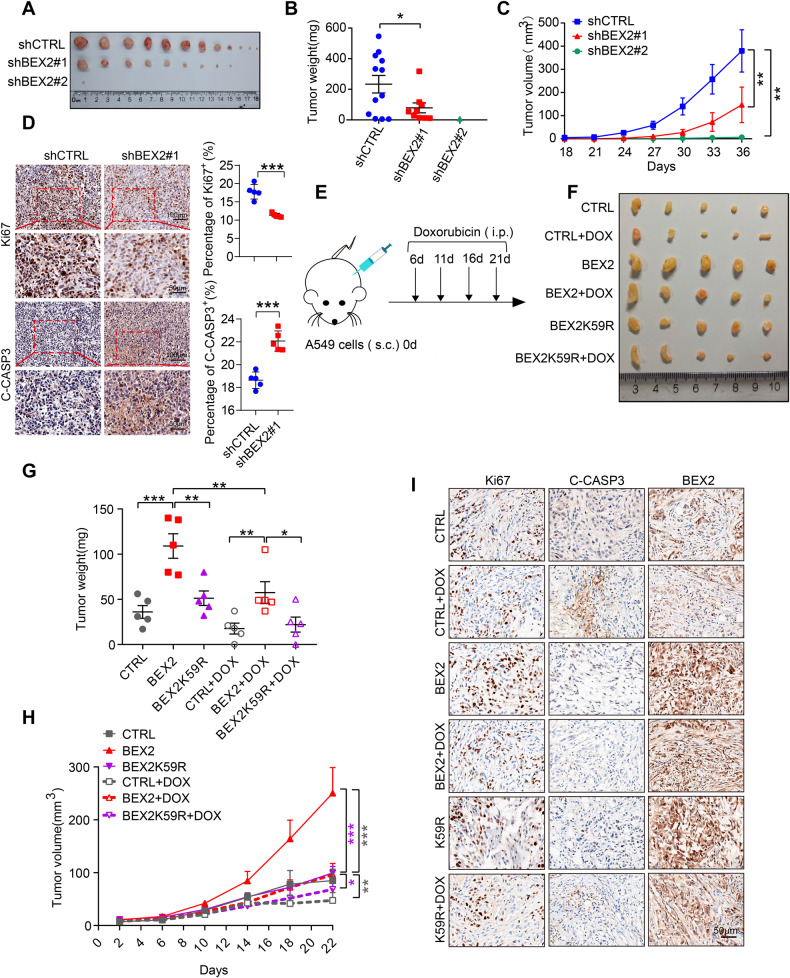
BEX2 promotes tumor growth in vivo. **A**–**C** BALB/c nude mice were injected subcutaneously with stably transduced H1299 cells. The transplanted tumors were removed and photographed (**A**). Tumors were isolated, and the weight (**B**) and growth (**C**) were measured. **D** Representative immunohistochemical staining for Ki67 and cleaved CASP3 in tumor tissues obtained from each experimental group. Dot plots show the mean value for the percentage of Ki67 and cleaved CASP3-positive cells with statistical evaluation (*n* = 4). Scale bars: 50 μm. **E** Schematic showing the model of xenografts. A549^NC^, A549^BEX2^ or A549^BEX2K59R^ cells were injected subcutaneously into the BALB/c nude mice (*n* = 5 per group). After tumor inoculation for 6 days, doxorubicin (3 mg/kg) was intraperitoneally injected at intervals of 4 days. The control groups were injected with saline. After 21 days, the mice were then euthanized. **F**–**H** The transplanted tumors were removed and photographed (**F**). Tumors were isolated, and the weight (**G**) and growth (**H**) were measured. **I** Immunohistochemistry analysis showing Ki67, cleaved CASP3 and BEX2 expression in the indicated tumor tissues. Scale bar: 50 μm. Data are presented as the mean ± SEM, and statistical significance was assessed by two-way ANOVA (**B**–**D**, **G**, **H**), *P < 0.05, **P < 0.01, ***P < 0.001.

Next, we decided to explore the role of BEX2 in tumor growth. H1299 cells were infected with BEX2 shRNA or control shRNA lentivirus. Then, the aforementioned H1299 stable clones were inoculated subcutaneously into BALB/c nude mice. The results showed that depletion of BEX2 strikingly inhibited tumor growth (Fig. 6A–C). Immunohistochemistry results showed that BEX2 knockdown decreased the abundance of Ki67-positive cells and caused a significant increase in immunoreactivity for cleaved CASP3, indicative of apoptosis (Fig. 6D). Overall, we confirmed that BEX2 inhibits apoptosis and promotes tumor growth.

To further verify the regulation of BEX2 on doxorubicin-induced apoptosis in vivo, A549 cells overexpressing BEX2 were subcutaneously injected into BALB/c nude mice to generate NSCLC xenograft models. Our results showed that A549-BEX2 cells exhibited a notable tumor-accelerative effect in the mouse model, which was consistent with the results mentioned above. Doxorubicin significantly inhibited the growth rate and weight of tumors, while overexpression of BEX2 abolished the inhibitory effects of doxorubicin (Fig. 6E–H). IHC results also showed that CASP3 apoptotic signaling was activated in the doxorubicin treatment group but inhibited in the BEX2 overexpression group (Fig. 6I). Together, these results demonstrate that BEX2 inhibits doxorubicin-induced cancer cell death in vivo.

Our data also showed that BEX2K59R significantly inhibited the growth rate and weight of tumors, compared with BEX2, and this effect was more obvious after doxorubicin treatment (Fig. 6F–H). Analyses of tumor tissues by IHC also showed that overexpression of BEX2K59R decreased the abundance of Ki67-positive cells compared with the WT group and caused an obvious enhancement in immunoreactivity for cleaved CASP3 (Fig. 6I). Together, these results show that crotonylation of BEX2 is required for inhibiting doxorubicin-induced apoptosis in vivo.

**Fig. 7:**
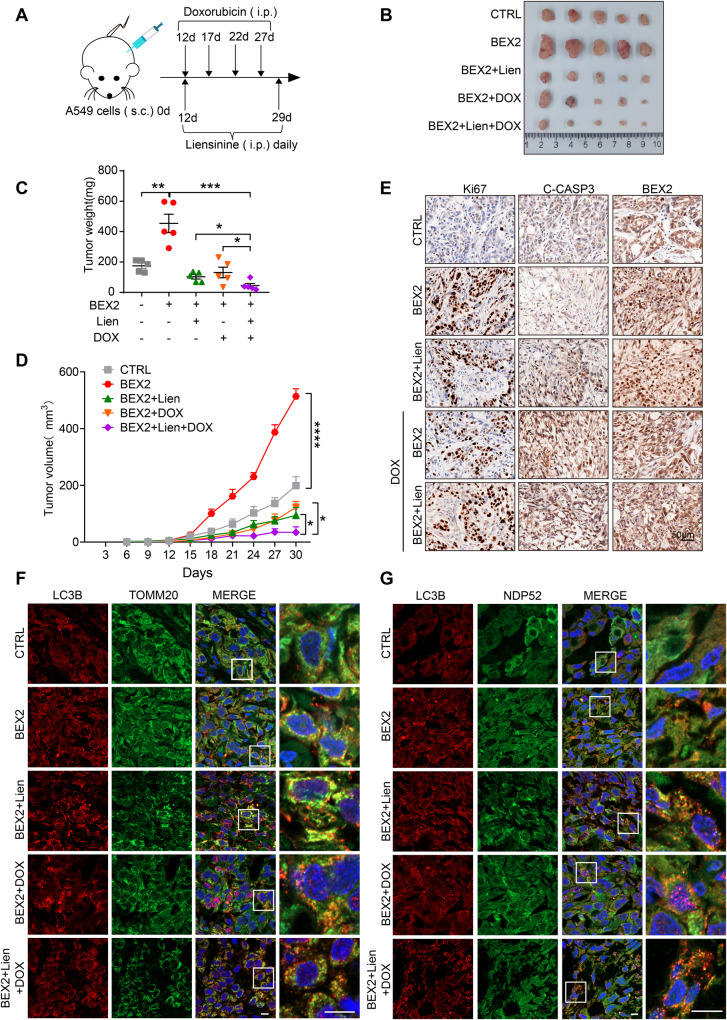
BEX2-regulated mitophagy promotes tumor growth in vivo. **A** Schematic showing the model of xenografts. BALB/c nude mice were injected subcutaneously with stably transduced A549^NC^ or A549^BEX2^ cells. After 6 days, mice were treated with liensinine (Lien, 60 mg/kg) daily and doxorubicin (DOX, 2 mg/kg) were intraperitoneally injected at intervals of 4 days. After 30 days, the mice were then euthanized. **B**–**D** The transplanted tumors were removed and photographed (**B**). Tumors were isolated, and the weight (**C**) and growth (**D**) were measured. **E** Immunohistochemistry analysis showing Ki67, cleaved CASP3 and BEX2 expression in the indicated tumor tissues. Scale bar: 50 μm. **F** Representative immunofluorescence staining detection for determination of the co-localization of LC3B and TOMM20 by confocal microscopy. Scale bars: 10 μm. **G** Representative immunofluorescence staining detection for determination of the co-localization of LC3B and NDP52 by confocal microscopy. Scale bars: 10 μm. Data are presented as the mean ± SEM, and statistical significance was assessed by two-way ANOVA (**C**, **D**), *P < 0.05, **P < 0.01, ***P < 0.001, ****P < 0.0001.

To further assess the role of BEX2-regulated mitophagy in apoptosis inhibition, A549-BEX2 cells were subcutaneously injected into BALB/c nude mice to generate NSCLC xenograft models. Liensinine was previously shown to inhibit late-stage mitophagy through blocking mitophagosome-lysosome fusion, and the combination of liensinine with doxorubicin increased the number of mitophagosomes [22]. Following treatment with doxorubicin and liensinine, the tumor weight and tumor growth rate were measured to evaluate the role of BEX2-mediated mitophagy in doxorubicin resistance (Fig. 7A). We found that liensinine treatment alone inhibited BEX2-induced tumor growth, as evidenced by the reduced weight and growth rate of tumors. Moreover, the BEX2 + DOX+Lien group further inhibited tumor growth compared to the BEX2 + DOX group (Fig. 7B–D). We were using immunohistochemistry to detect the expression of Ki67, cleaved-CASP3 and BEX2. The results also verified that BEX2 promoted Ki67 expression and inhibited the activation of CASP3 apoptotic signaling. However, liensinine treatment abolished the protective role of BEX2 in the apoptotic signaling pathway, as evidenced by increased cleaved-CASP3 expression (Fig. 7E).

Immunofluorescence microscopy analysis was employed to confirm whether BEX2 inhibits doxorubicin-induced apoptosis via regulating mitophagy in vivo. Overexpression of BEX2 increased the mitophagosome number measured by LC3B puncta and co-localization of LC3B with TOMM20 compared to control, indicating that BEX2 indeed induced mitophagy. The combination of liensinine with doxorubicin augmented the role of BEX2 to increase the number of LC3B puncta and the co-localization of LC3B with TOMM20 compared to liensinine or doxorubicin treatment alone (Fig. 7F). Our earlier findings have implied that BEX2 promotes the interaction of NDP52 and LC3B to regulate mitophagy in vitro. Immunofluorescence microscopy was performed to test this possibility in vivo to evaluate the co-localization of NDP52 and LC3B. Interestingly, overexpression of BEX2 promotes the co-localization of NDP52 and LC3B. The co-localization was further strengthened under liensinine and doxorubicin combination treatment compared with single drug (Fig. 7G). Thus, these data indicate that BEX2-regulated mitophagy plays an important role in inhibiting apoptosis in vivo.

### BEX2 is upregulated in lung adenocarcinoma, and high BEX2 expression level is associated with poor prognosis in lymph node metastasis-free cancer

**Fig. 8:**
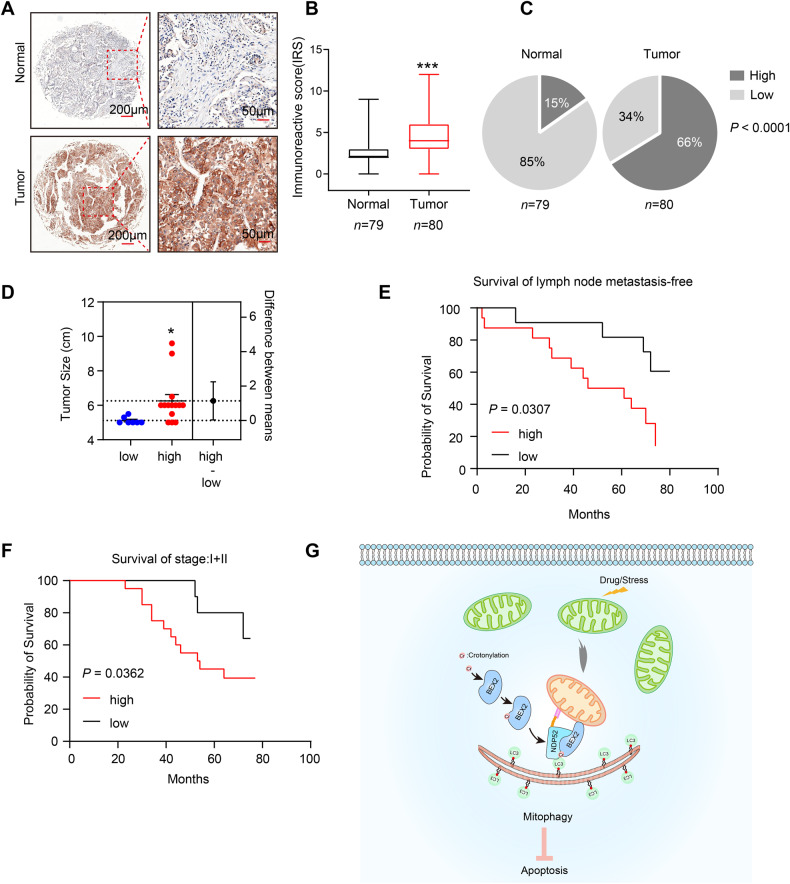
BEX2 is upregulated in lung adenocarcinoma and high BEX2 expression level is associated with poor prognosis in lymph node metastasis-free cancer. **A** Representative IHC images showed the protein levels of BEX2 in TMA. **B** Immunoreactivity scores (IRS) of BEX2 in normal and tumor tissues shown as a box plot. *** *P* < 0.001. **C** Percentages of low and high expression of BEX2 in normal and tumor tissues are shown as a pie chart. *P* < 0.0001. **D** Tumor size (>5 cm) of low and high expression of BEX2 in LUAD tissues are shown as a dot plots. *P < 0.05. **E**, **F** Survival of lymph node metastasis-free was compared between LUAD patients with low and high level of BEX2 (*n* = 11, low BEX2 levels; *n* = 16, high BEX2 levels) (**E**). Survival of clinical stage (I + II) was compared between LUAD patients with low and high level of BEX2 (*n* = 13, low BEX2 levels; *n* = 27, high BEX2 levels) (**F**). Survival data were analyzed by the Kaplan-Meier method and log-rank test. **G** Schematic diagram of BEX2-mediated mitophagy activation to inhibit apoptosis in NSCLC cells.

To determine whether our findings have potential clinical implications, tissue microarrays (TMA) containing 80 lung adenocarcinoma (LUAD) cases and 79 adjacent normal tissue cases from patients were used to examine the expression of BEX2. The IHC staining results were quantified as described in the methods section, and we observed high cytoplasmic BEX2 levels in LUAD samples compared to normal tissues (Fig. 8A, B). In tumor tissue, 53 (66.3%) of the 80 specimens showed high expression of cytoplasmic BEX2. While in adjacent normal tissue, 12 (15.2%) of the 79 specimens showed high expression, indicating that there were more tumor tissues with high expression of cytoplasmic BEX2 than adjacent normal tissues (Fig. 8C). Notably, cytoplasmic BEX2 expression was significantly associated with T-primary tumors and the age of the patients (Supplementary Table 1). We further assessed the relationship between the expression level of BEX2 and tumor size (>5 cm). As shown in Fig. 8D, cases with high cytoplasmic expression of BEX2 had larger tumors. Moreover, high expression of cytoplasmic BEX2 correlated with lower survival in lymph node metastasis-free cases (Fig. 8E). Besides, high cytoplasmic BEX2 levels were positively correlated with a poor prognosis in clinical stage (I + II) cases (Fig. 8F). Taken together, these results demonstrate that BEX2 is overexpressed in lung adenocarcinoma and is associated with poor prognosis in lymph node metastasis-free patients and clinical stage (I + II) patients.

## Discussion

In the present study, we proposed that cytoplasmic BEX2 was involved in the regulation of mitophagy. Our data showed that crotonylated BEX2 promoted mitophagy, which played a crucial role in inhibiting chemotherapy-induced apoptosis in NSCLC cancer cells and in vivo (Fig. 8G).

In the previous study, it is reported that BEX2 interacts with the transcription factor LMO2, NSCL2 or LDB1 in a DNA-binding complex that recognizes the E-box element and promotes transcription [3]. Recent studies have revealed that BEX2 may localize in cytoplasm and/or mitochondria [2, 4] and regulate apoptosis and tumor growth in cancer cells. However, the molecular mechanism underlying its roles in the cytoplasm and/or mitochondria of cancer cells remains unclear. In the present study, we focused on the role of BEX2 in the cytoplasm of NSCLC cells. We found that BEX2 promoted NDP52-mediated mitophagy. Moreover, the expression of BEX2 was increased after anticancer drug treatment. And we showed that cell viability increased in cells expressing BEX2 after anticancer drug treatment. In addition, its overexpression inhibited chemotherapy-induced apoptosis, and BEX2 inhibited chemotherapy-induced apoptosis via promoting NDP52-mediated mitophagy.

To date, the function of BEX2 remains poorly understood, and it has been suggested that BEX2 might be localized to the nucleus and act as a regulator during embryonic development to modulate transcriptional activity with LMO2, NSCL2, and LDB1 [3]. BEX2 has been reported to suppress mitochondrial function through its interaction with the TUFM mitochondrial protein [2]. Moreover, BEX2 has been found to promote the reprogramming of fibroblasts into pluripotent stem cells, dependent on reduced mitochondrial ATP production [40]. Furthermore, BEX2 has been identified as a pseudosubstrate inhibitor of CUL2^FEM1B^ that binds to the substrate binding pocket of FEM1B and inhibits FNIP1 ubiquitination without further modification, which protects cells from unwarranted ROS accumulation [4]. The detailed function of BEX2 in mitochondria is still unclear. In the present study, we verified that BEX2 is also localized to mitochondria. Mitophagy is a mechanism by which mitochondria are degraded via a selective form of autophagy [41]. Our findings indicated that the localization of BEX2 to mitochondria increased after mitophagy inducer CCCP treatment. BEX2 can bind to NDP52 through the CC domain of NDP52. In addition, BEX2 promoted the interaction of NDP52 and LC3B in CCCP-induced mitophagy. As it is well known, mitophagy regulators, such as PINK1/Parkin, BNIP3, and NIX (known as BNIP3L) have independent roles in mitophagy regulation. In PINK1/Parkin-dependent mitophagy, NDP52 and OPTN are preferential adaptors [18, 23]. In the present study, our results revealed a novel pathway for BEX2-mediated mitophagy. In CCCP-induced PINK1/Parkin-dependent mitophagy, PINK1 recruits Parkin to the mitochondria and then carries out the ubiquitination and degradation of certain mitochondrial outer membrane proteins. It is reported that Parkin has been shown to be downregulated in multiple cancer cell lines and primary tumors, including lung carcinoma [19, 42, 43]. Meanwhile, our results also showed that BEX2 can enhance mitophagy in NSCLC cells lacking Parkin expression [35], indicating that other E3 ligases such as TRAF2 etc. might be involved in BEX2-induced mitophagy, which requires further experimental verification in the future. In summary, BEX2 is localized to mitochondria and enhances the interaction of NDP52 with LC3B, thereby enhancing mitophagy in NSCLC cells.

Mitophagy is crucial in regulating cancer cell death [41, 44, 45]. Our results suggest that BEX2-regulated mitophagy enhances the resistance of NSCLC cells to apoptosis in vitro and vivo. We demonstrated that inhibition of BEX2-regulated mitophagy sensitized tumor cells to apoptosis, suggesting that mitophagy plays a protective and pro-survival role in these cells. When we used mitophagy inhibitors or siRNAs to knock down the expression of ATG5 or ATG7 in BEX2-overexpressed cells, BEX2 overexpression-induced apoptosis inhibition was impaired. And we found that NDP52 knockdown reversed BEX2-induced apoptosis inhibition, which proved that BEX2 inhibited apoptosis via activation of NDP52-dependent mitophagy. Consistently with BEX2, ARIH1-induced mitophagy has been found to enhance the resistance of cancer cells to chemotherapy-induced cell death [35]. Regarding the mechanism of the tumor-promoting role of BEX2, we showed that BEX2 promoted tumor growth and inhibited doxorubicin-induced apoptosis in vivo, which was reversed by inhibiting mitophagy with liensinine. Notably, combined with our present in vivo findings, BEX2-induced mitophagy could be considered a potential target for chemotherapy resistance in NSCLC treatment.

An increasing number of proteomics studies have expanded the crotonylation substrates to include non-histone proteins. However, the mechanisms of non-histone protein crotonylation remain to be explored. BEX2, a non-histone protein, was found to have crotonylation at K59. Interestingly, we found that K59 crotonylation is required for enhancing the binding of NDP52 and LC3B and the regulation of mitophagy. Moreover, our studies showed that crotonylation of BEX2 plays a crucial role in enhancing mitophagy and inhibiting apoptosis. The K59R mutation of BEX2 inhibits mitophagy by affecting the interaction of NDP52 and LC3B. In addition, the K59R mutation of BEX2 sensitizes tumor cells to apoptosis. However, the detailed effect of crotonylation on the function of BEX2 needs to be further investigated.

Clinical investigation of the role of BEX2 in patients remains unclear. In hepatocellular carcinoma, BEX2 induces dormant cancer stem cell properties and results in poor prognostic of patients [6]. Our study found that cytoplasmic BEX2 is highly expressed in LUAD, and cases with high expression of cytoplasmic BEX2 had larger tumors. In lymph node metastasis-free lung adenocarcinoma, high expression of cytoplasmic BEX2 correlated with lower survival, and the same result was obtained in stage (I + II). Although the number of cases is limited, this result may suggest that BEX2 can be used as a marker for the potential benefit of LUAD in a certain stage.

In summary, we elucidated that BEX2 promotes mitophagy in NSCLC cells and is involved in the protection of these cells from apoptosis. In the past two decades, the use of small molecule tyrosine kinase inhibitors and immunotherapy has resulted in great achievements in selected patients. However, the overall cure and survival rates for NSCLC remain low. Therefore, continued research into new drugs, new targets, and combination therapies is required to improve the outcomes of NSCLC patients [46]. Thus, based on our present findings, we propose that combination treatment with mitophagy inhibitors and anticancer drugs that target BEX2 may represent a potential strategy for NSCLC treatments.

## Materials and methods

### Cell lines and cell culture

The HEK293FT cell line was cultured in Dulbecco’s modified Eagle’s medium (DMEM, 3.7 g NaHCO_3_/L, Sigma-Aldrich) supplemented with 10% (V/V) fetal bovine serum (FBS, TOCYTO). H1299, H157, H1792 and A549 cell lines were cultured in RPMI 1640 2.0 (1640, 2.0 g NaHCO_3_/L, Sigma-Aldrich) supplemented with 10% FBS. All cell lines were originally obtained from the American Type Culture Collection (ATCC) and were maintained at 37 °C in a humidified atmosphere consisting of 5% CO_2_ and tested to ensure no mycoplasma contamination.

### Reagents and antibodies

The chemicals used in our experiments were CCCP (HY-100941), liensinine (HY-N0484), valinomycin (HY-N6693), and cisplatin (HY-17394), which were purchased from MedChemExpress. Doxorubicin (S1208) were purchased from Selleckchem. Pemetrexed (SML1490) was purchased from Sigma-Aldrich.

The primary antibodies used in the western blot and immunoprecipitation assays were as follows: anti-ACTB (A1978), anti-GFP (G1544), anti-FLAG (F7425, F1804), anti-GAPDH (G8795), and anti-MYC (C3956, M4439) were obtained from Sigma-Aldrich; anti-LC3B (2775), anti-ATG5 (9980), anti-acetylated lysine (6952),anti-CASP8 (9746), anti-Parkin (4211), ATG7 (8558) were obtained from Cell Signaling Technology; anti-TIMM23 (11123-1-AP), anti-TOMM20 (11802-1-AP), anti- MFN1 (66776-1-Ig), and anti-COX4 (11242-1-AP) were obtained from Proteintech; anti-BEX2 (SC-398486), anti-HSP60 (SC-13115), and anti-NDP52 (SC-376540) were obtained from Santa Cruz Biotechnology; anti-CASP3 (NB100-56708) was obtained from Novus Biologicals; anti-PARP-1 (556494) was obtained from BD Biosciences; and anti-pan-Kcr (PTM-502) was obtained from PTM Biolabs.

### Plasmids and siRNA transfection

The cells were transfected with jetPRIME transfection reagent (PolyPlus-transfection) or LipoMax reagent (Sudgen Biotechnology) in serum-free Opti-MEM (Gibco) according to the instruction manual. All siRNAs were synthesized by GenePharma (Shanghai, China). BEX2#1 and BEX2#2 siRNA target sequences: 5′-GCCCTACCTTTGAATGTTA-3′ and 5′-GGAGCAAGTTGCTAATAAA-3′, respectively. ATG5 siRNA target sequences: 5′-CCTTTGGCCTAAGAAGAAA-3’. ATG7 siRNA target sequences: 5′-GGAGTCACAGCTCTTCCT-3′. NDP52 siRNA target sequences: 5′-GGAACTGAAAGTGAAAGAA-3′. BEX2 3′-UTR siRNA target sequences: 5′- GGTGTACCTTTGTCGTAAA-3′.

The GFP-Parkin plasmid was a gift from Dr. Zhiyin Song. Human BEX2 and BEX2 mutations (BEX2K59R, BEX2 LIR, BEX2 Y97A/W99A), NDP52 and NDP52 mutations (SKICH, CC, LIM-L) coding regions were amplified from cDNA and subcloned into pcDNA3.1; HA-Parkin were amplified from GFP-Parkin and subcloned into pcDNA3.1.

### Western blot and immunoprecipitation

The cells were harvested and lysed in lysis buffer on ice for 30 min and then purified via centrifugation at 13,200 rpm for 15 min at 4 °C. Equivalent protein quantities were subjected to 10–14% SDS-PAGE, transferred to PVDF membranes, and probed with primary antibodies, followed by the appropriate peroxidase-conjugated secondary antibodies (anti-mouse, AP124P; anti-rabbit, AP307P). A chemiluminescence kit (WBKLS0500) was used to visualize the immunoreactive bands. Images were acquired with Amersham Imager 680 (GE).

For immunoprecipitation, cells were transiently transfected with the indicated plasmids for a specified time. Cells were collected and lysed in IP lysis buffer for 30 min and then purified via centrifugation at 13,200 rpm for 15 min at 4 °C. FLAG M2 beads (A2220, Sigma-Aldrich), MYC beads (E6654, Sigma-Aldrich), and glutathione sepharose beads (16100, Invitrogen) were incubated with the clarified lysates at 4 °C for 6 h, or the clarified lysates were immunoprecipitated with a specific antibody and protein A-sepharose (101142, Invitrogen) or protein G-sepharose (101243, Invitrogen) for 4 h at 4 °C. The immune complexes were washed 3 times with IP lysis buffer, and the bead-conjugated proteins were denatured in 2× SDS loading buffer for 10 min at 100 °C. Samples were then separated by SDS-PAGE and detected by immunoblotting.

### Mt-Keima Mitophagy assay

Mt-Keima mitophagy assays were performed as previously described [17]. Briefly, A549 or H1299 cells were co-transfected with HA-Parkin and mt-Keima for 48 h, and then the cells were cultured in glass-bottom dishes. Next, A549 cells were transfected with BEX2 for 8 h or H1299 cells were transfected with BEX2 siRNA for 24 h, followed by incubation with CCCP (20 μM) for 6 h and subsequent analysis by confocal microscopy. The cells were scanned, and images were collected using a ZEISS LSM 900 confocal microscope (×63 oil). Ratiometric analysis was performed using ImageJ software. Mt-Keima is a variant of RFP that is targeted to the mitochondrial matrix. Its fluorescence changes in response to pH (green, 488 nm, pH = 7; red, 561 nm, pH = 4).

### Immunofluorescence microscopy

Immunofluorescence microscopy assays were performed as previously described [47]. Briefly, cells were fixed and permeabilized in PHEMO buffer (0.025 M HEPES, 0.068 M PIPES, 0.003 M MgCl_2_·6H_2_O, 0.015 M EGTA·Na_2_, 10% DMSO, pH=6.8). additional reagents were added before use, with final concentrations of 0.05% glutaraldehyde, 0.5% Triton X-100, and 3.7% formaldehyde, and then incubated at room temperature for 10 min. After being washed with PBS three times, cells were blocked in 3% BSA and then stained using primary antibodies overnight at 4 °C. After being washed with PBS three times, the secondary antibodies used were anti-rabbit Alexa Fluor 555 dye conjugate (A27039, invitrogen) or anti-mouse Alexa Fluor 488 dye conjugate (A28175, invitrogen) for 1 h at room temperature in the dark and subsequently washed with PBS three times. Nuclei were stained with DAPI (Sigma-Aldrich) for 5 min. Finally, the cells were washed with PBS three times. After mounting, the cells were visualized using a confocal microscope (ZEISS, LSM 880, 63× oil). Analysis was performed using ImageJ software.

### CCK-8 assay

Cells were seeded in 96-well culture plates at 6,000, and then cells were cultured for 24 h. After treatment, the medium was removed, and 100 μL of CCK8 mixture (10 μL of CCK8 reagent (Meilunbio, MA0218) + 90 μL of culture medium) was added to each well and incubated for 1.5 h at 37 °C. The absorbance of each well was measured at 450 nm. All experiments were repeated at least three times.

### Flow cytometry analysis

Annexin V-FITC Apoptosis Detection Kit (Biobox Biotech, BA111000) and 7-AAD Cell Viability Assay kit (Beyotime, C1053S) were used for cell apoptosis analysis according to the protocol.

### Mitochondria isolation

Briefly, cells were collected and washed with PBS, and then, mitochondria isolated according to Cytoplasmic and Mitochondrial Protein Extraction Kit (Sangon Biotech, C5000511).

### Lentivirus infection

The sequences of lentiviral shBEX2: 5′-CCTTTGAATGTTAGTGAATAC-3′ and 5′- GCCCTACCTTTGAATGTTAGT -3′. The shBEX2 sequences ligated into the pLKO.1 vector. BEX2 and BEX2 mutations subcloned into lentiviral expression vector. Lentivirus-expressed vector was produced in HEK293FT cells packaged by pMD2.0 G and psPAX2. For stable infection, cells were plated in 6-well plates in 2 ml of medium. After overnight incubation, the medium was removed and replaced with 1 ml per well of medium containing 8 μg/ml polybrene. Then, 1 ml of retroviral particles was added into each well. After 24 h incubation, the medium was refreshed with RPMI 1640 complete medium containing 2 μg/ml puromycin or 2 μg/ml blasticidin. The medium was refreshed every 2–3 days for 2 weeks. The transfection efficiency was confirmed using western blot.

### Xenograft assay

Female BALB/c nude mice (4–5 weeks old) were purchased from Vital River Laboratories (Beijing, China) and kept in pathogen-free conditions.

A total of 1.5 × 10^6^ H1299 cells (H1299-shNC, H1299-shBEX2#1, H1299-shBEX2#2) were injected subcutaneously into the mice. Mouse weight and tumor size were measured every three days.

A total of 1.5 × 10^6^ A549 cells (A549-NC, A549-BEX2) were injected subcutaneously into the mice. After tumor inoculation, doxorubicin (3 mg/kg) was intraperitoneally injected at intervals of 4 d. The control groups were injected with saline. Mouse weight and tumor size were measured every second day.

A total of 1.5 × 10^6^ A549 cells (A549-NC, A549-BEX2) were injected subcutaneously into the mice. After tumor inoculation, liensinine (60 mg/kg) was administered daily by intraperitoneal injection, and doxorubicin (2 mg/kg) was injected at intervals of 4 d. The control groups were injected with saline. Mouse weight and tumor size were measured every three days.

Tumor volume was calculated using the formula π/6 × length × width^2^. The tumors were extracted and weighed when the length of the tumor reached 1 cm. All tumors were excised and either formalin-fixed or flash-frozen at −80 °C until further use.

### Immunohistochemistry and immunofluorescence (IF) staining

Tumor xenografts were fixed in 4% formaldehyde overnight at room temperature and embedded in paraffin. Sections of 4 μm in thickness were used for immunohistochemistry and immunofluorescent staining. Samples were baked at 55 °C for 4 h, then de-paraffinized by for 10-min extractions in xylene, following by 5-min each of descending grade of alcohol (100%, 100%, 90%, 70%, 30%). For immunohistochemistry, sections were pre-treated with 3% hydrogen peroxide for 15 min before antigen retrieval. antigen retrieval (Tris-EDTA buffer) was performed in a microwave oven for 10 min. Next, to avoid nonspecific binding, the samples were incubated with normal goat serum at RT for 30 min and probed with primary antibodies at 4 °C overnight. After being washed with PBS three times, followed by incubation with biotin-conjugated secondary antibody (SP-900, ZSGB-BIO) at RT for 20 min. DAB (ZLI-9018, BIO) was used as chromogen and nuclei were counterstained with hematoxylin. The quantitative results for IHC images were calculated by ImageJ software.

For immunofluorescence staining, sections were incubated with Alexa Fluor-488 and Alexa Fluor-555-conjugated secondary antibodies (invitrogen), mounted with DAPI (1 μg/ml, Sigma-Aldrich) to stain nucleus, and visualized using a confocal microscope (ZEISS, LSM 900, 63× oil).

The primary antibodies are listed the below: anti-Ki67 (9449), anti-cleaved-CASP3 (9661) were obtained from Cell Signaling Technology; anti-BEX2 (12390-1-AP), anti-TOMM20 (66777-1-Ig), anti-LC3B (18725-1-AP) were obtained from Proteintech; anti-NDP52 (SC-376540) were obtained from Santa Cruz Biotechnology.

### Tissue microarrays (TMA) and scoring

LUAD tissue microarray (catalog LUC1601) was purchased from Superbiotek Inc. TMA involved matched normal tissues as well as tumor tissue samples from 80 patients. After Immunohistochemistry (IHC) staining, the TMA chips of human LUAD samples were digitally scanned by the automated slide scanner, and the whole field of each tissue spot was obtained for IHC evaluation. The expression levels of BEX2 was scored semiquantitatively based on staining intensity and distribution using the immunoreactive score (IRS). Briefly, IRS (immunoreactive score) = SI (staining intensity) × PP (percentage of positive cells). SI was assigned as: 0=negative; 1=weak; 2=moderate; 3=strong. PP is defined as 0 = 0%; 1 = 0–25%; 2 = 25–50%; 3 = 50–75%; 4 = 75–100%. For categorization of the continuous IRS values into low and high, we chose a cutoff point for the measurements (range 0–12, cut point ≤ 4 versus > 4).

### Statistics

GraphPad Prism 8.0.1 software was used for the statistical analysis. All data are presented as the mean ± SD. For the xenograft assays, the data are presented as the mean ± SEM. Differences between groups were identified using two-sided Student’s *t*-test and one-way or two-way ANOVA. The χ^2^ test was applied for categorical variables. *P* < 0.05 was considered statistically significant. Statistical significance was also taken as *P < 0.05, **P < 0.01, ***P < 0.001 and ****P < 0.0001.

### Reporting summary

Further information on research design is available in the Nature Research Reporting Summary linked to this article.

### Supplementary information

supplementary Figure 1-8

supplementary Table 1

Original western blots

Reporting Summary

## Data Availability

All data generated or analysed during this study are included in this published article and its supplementary information files.
