# Supplementary material for: Crotonylated BEX2 interacts with NDP52 and enhances mitophagy to modulate chemotherapeutic agent-induced apoptosis in non-small-cell lung cancer cells
Source: Cell Death Dis. 2023 Sep 30;14(9):645. doi: 10.1038/s41419-023-06164-6 (PMC10542755; doi:10.1038/s41419-023-06164-6)
Supplement: Supplementary file 1 — supplementary Figure 1-8 [file 41419_2023_6164_MOESM1_ESM.docx]

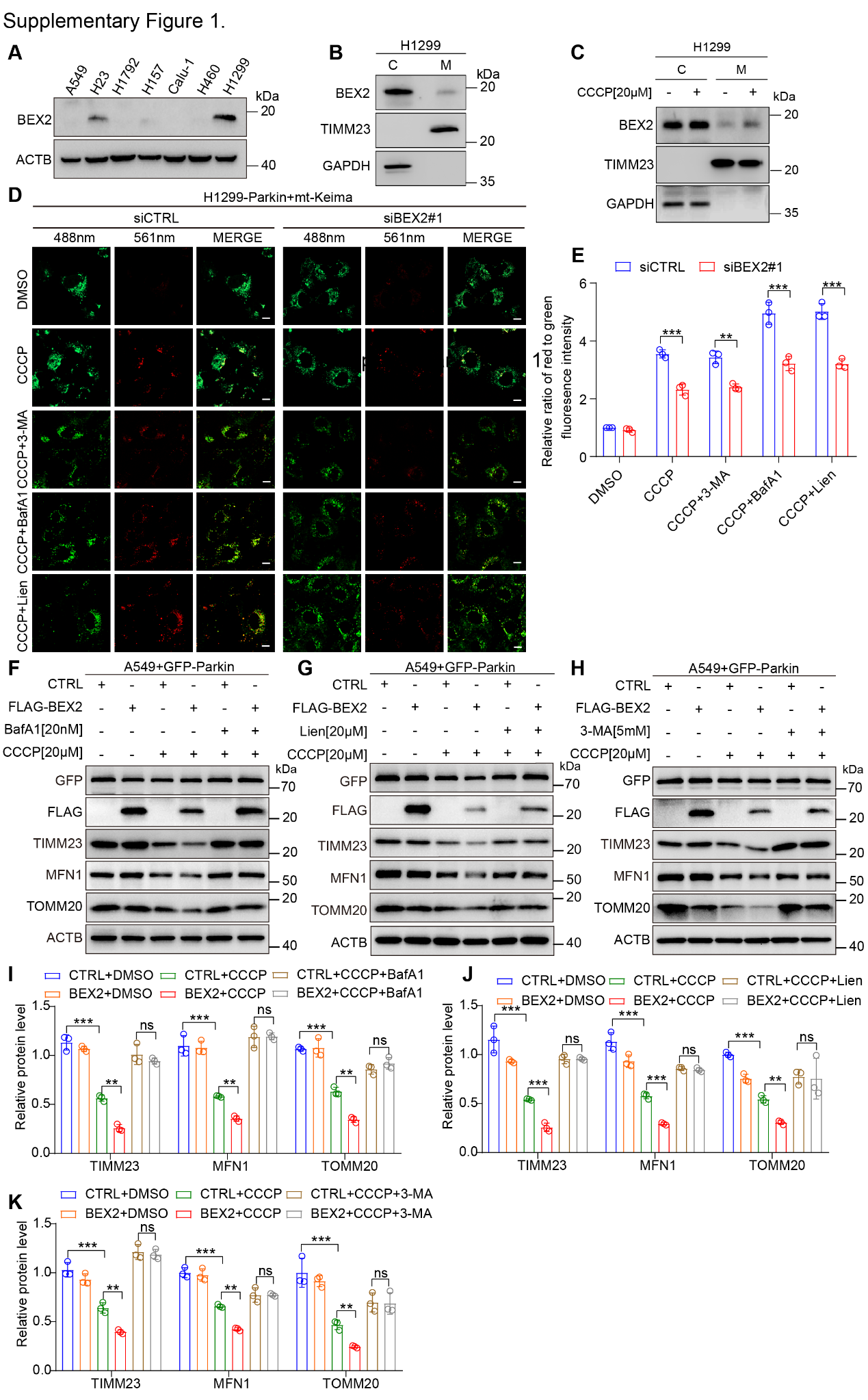


**Supplementary Figure 1**. **BEX2 promotes mitophagy in NSCLC cells.**

**(A)**. Western blot of BEX2 from seven non-small cell lung cancer cells.

**(B)**. H1299 cells were used to isolate mitochondria and then analyzed by western blotting with anti-BEX2, anti-TIMM23 (mitochondrial markers), and anti-GAPDH (cytosolic marker) antibodies. C, cytosol; M, mitochondria.

**(C)**. H1299 cells were incubated with CCCP (20 μM) for 6 h, and then mitochondria were isolated and analyzed by western blotting with anti-BEX2, anti-TIMM23 (mitochondrial markers), and anti-GAPDH (cytosolic markers). C, cytosol; M, mitochondria.

**(D, E)**. H1299 cells were co-transfected with HA-Parkin and mt-Keima, and then the cells were transfected with siCTRL or siBEX2. Cells were pre-treated with BafilomycinA1(20 nM), liensinine (20 μM), and 3-MA (5 mM) for 0.5h then treated with CCCP (20 μM) for 6 h. Mitophagy flux was monitored by confocal microscopy. Green indicates mt-Keima fluorescence excited at 488 nm (measuring mitochondria with a neutral pH), and red indicates mt-Keima fluorescence excited at 561 nm (measuring mitochondria with an acidic pH) (D). Quantification of the relative ratio of fluorescence intensity (561 nm: 488 nm) of the cells (E). Data are presented as the mean ± SD (*n* = 3 independent experiments, 20 cells per experiment), and statistical significance was assessed by two-way ANOVA. ***P*<0.01. Scale bars: 10 μm.

**(F)**. A549 cells were transfected with GFP-Parkin and CTRL(pcDNA3.1) or BEX2. Cells were pre-treated with BafilomycinA1(20 nM) for 0.5h and then incubated with CCCP (20 μM) for 6h. Cell lysates were analyzed by western blotting with the indicated antibodies.

**(G)**. A549 cells were transfected with GFP-Parkin and CTRL(pcDNA3.1) or BEX2. Cells were pre-treated with liensinine (20 μM) for 0.5h and then incubated with CCCP (20 μM) for 6h. Cell lysates were analyzed by western blotting with the indicated antibodies.

**(H)**. A549 cells were transfected with GFP-Parkin and CTRL(pcDNA3.1) or BEX2. Cells were pre-treated with 3-MA (5 mM) for 0.5h and then incubated with CCCP (20 μM) for 6h. Cell lysates were analyzed by western blotting with the indicated antibodies

**(I, J, K)**. The relative protein levels in F, G, and H were further evaluated by densitometry analysis using ImageJ software and quantified for the ratio of TIMM23/MFN1/TOMM20: ACTB. Data are presented as the mean ± SD (*n*=3 independent experiments), and statistical significance was assessed by one-way ANOVA. ns, not significant, ***P*<0.01, ****P*<0.001.


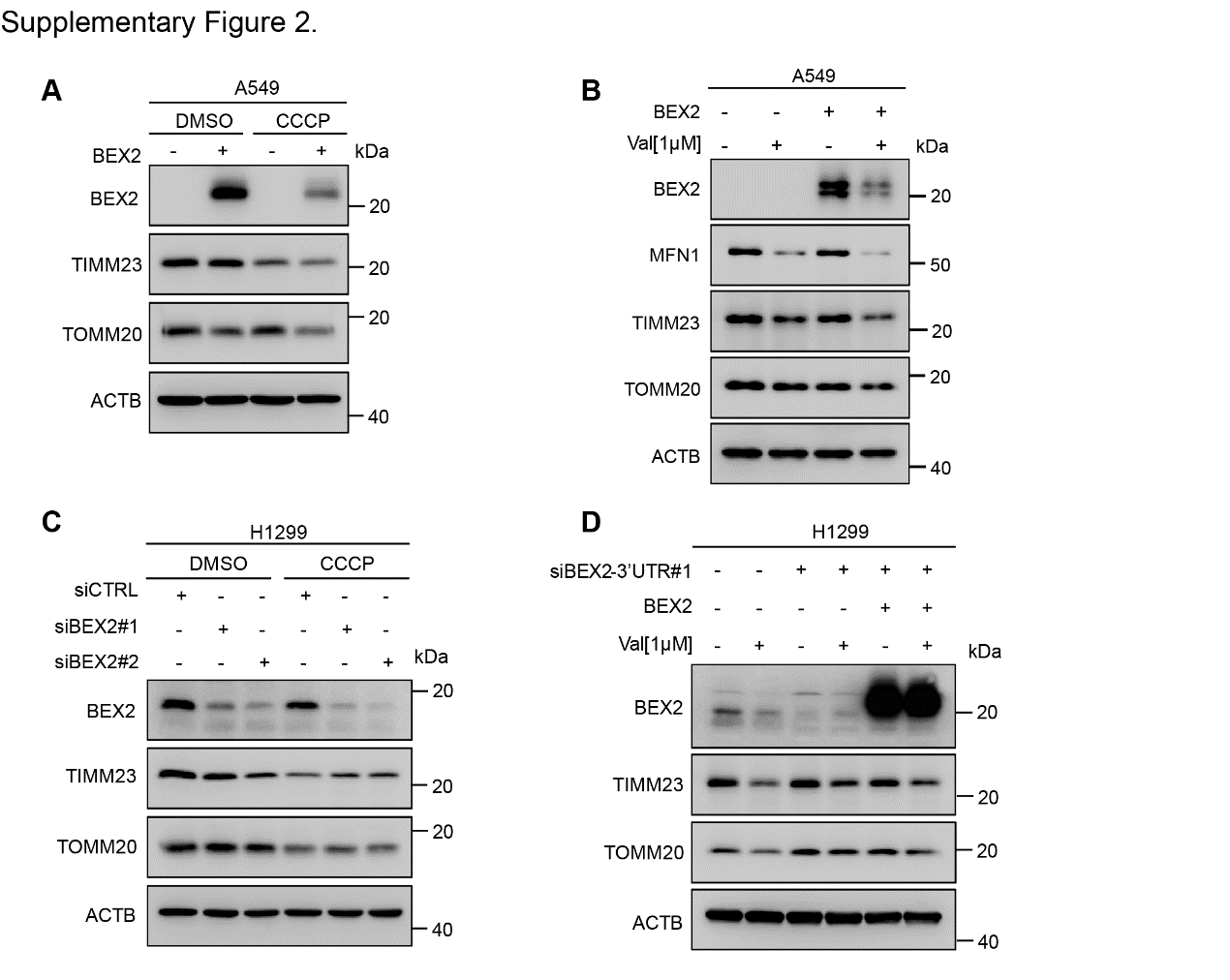


**Supplementary Figure 2**. **BEX2 promotes Parkin-independent mitophagy in NSCLC cells.**

**(A)**. A549 cells were transfected with CTRL(pcDNA3.1) or BEX2, and then the cells were treated with CCCP (20μM) for 6 h. Cell lysates were analyzed by western blotting with the indicated antibodies.

**(B)**. A549 cells were transfected with CTRL(pcDNA3.1) or BEX2. Then the cells were treated with valinomycin (1μM) for 6 h. Cell lysates were analyzed by western blotting with the indicated antibodies.

**(C)**. H1299 cells were transfected with siCTRL or siBEX2, and further incubated with CCCP (20 μM) for 6h. Cell lysates were analyzed by western blotting with the indicated antibodies.

**(D)**. H1299 cells were transfected with siCTRL or siBEX2, and then overexpressed CTRL(pcDNA3.1) or BEX2. Cells were further incubated with valinomycin (1 μM) for 6h. Cell lysates were analyzed by western blotting with the indicated antibodies.


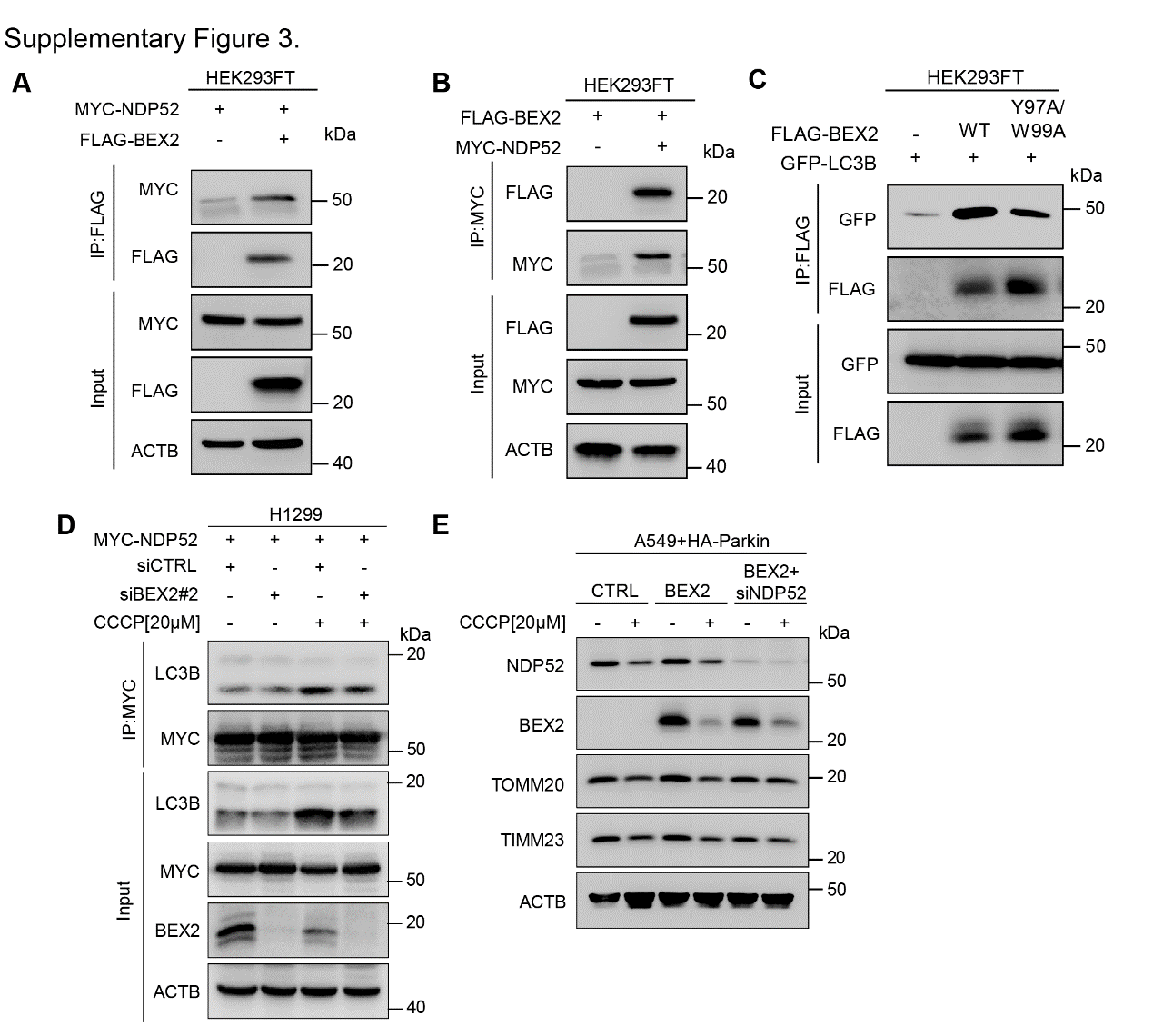


**Supplementary Figure 3**. **BEX2 interacts with NDP52 and LC3B to regulate mitophagy in NSCLC cells.**

**(A-B)**. HEK293FT cells were co-transfected with MYC-NDP52 and CTRL(pcDNA3.1) or FLAG-BEX2, and co-IP assays were carried out with FLAG antibody (A) or MYC antibody (B), followed by western blotting using the indicated antibodies.

**(C)**. HEK293FT cells were co-transfected with wild-type FLAG-BEX2 or FLAG-BEX2Y97A/W99A in the presence of GFP-LC3B, followed by IP-Western analyses.

**(D)**. H1299 cells were co-transfected with MYC-NDP52 and siCTRL or siBEX2#2, and then the cells were incubated with CCCP (20 μM) for 6 h. Cell lysates were incubated with an MYC antibody by co-IP assays followed by western blotting using the indicated antibodies.

**(E)**. A549 cells were transfected with HA-Parkin. Cells then were transfected with siCTRL or siNDP52 and CTRL(pcDNA3.1) or BEX2. The cells were treated with CCCP (20 μM) for 6h. Cell lysates were analyzed by western blotting with the indicated antibodies.


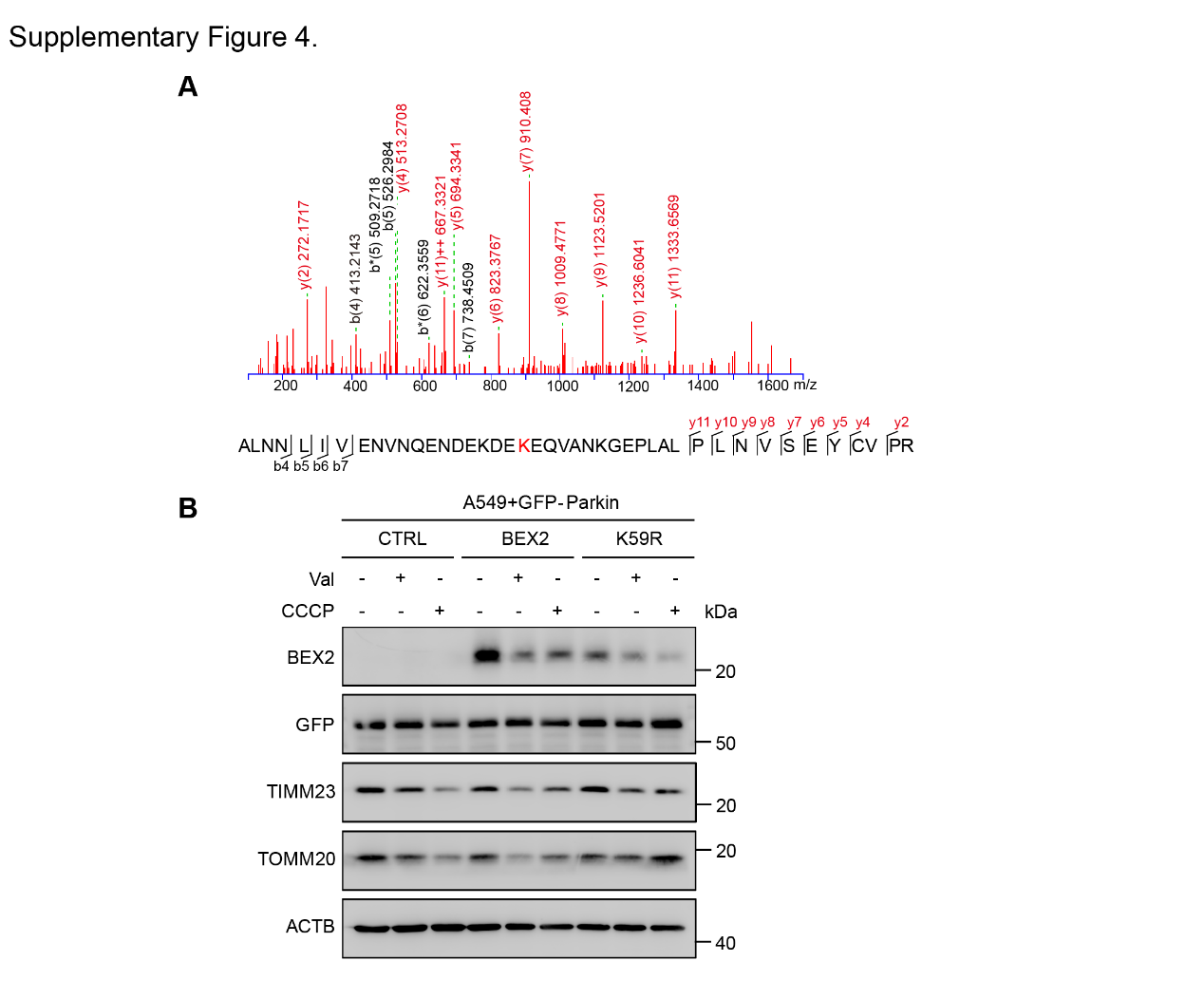


**Supplementary Figure 4. Crotonylation modification of BEX2 is required for the mitophagy regulation.**

**(A)** MS/MS spectra of the BEX2 peptide containing crotonylation-K59.

**(B)**. A549 cells were transfected with GFP-Parkin, BEX2 and BEX2K59R. Cells were treated with CCCP (20 μM) or valinomycin for 6 h. Cell lysates were analyzed by western blotting with the indicated antibodies.


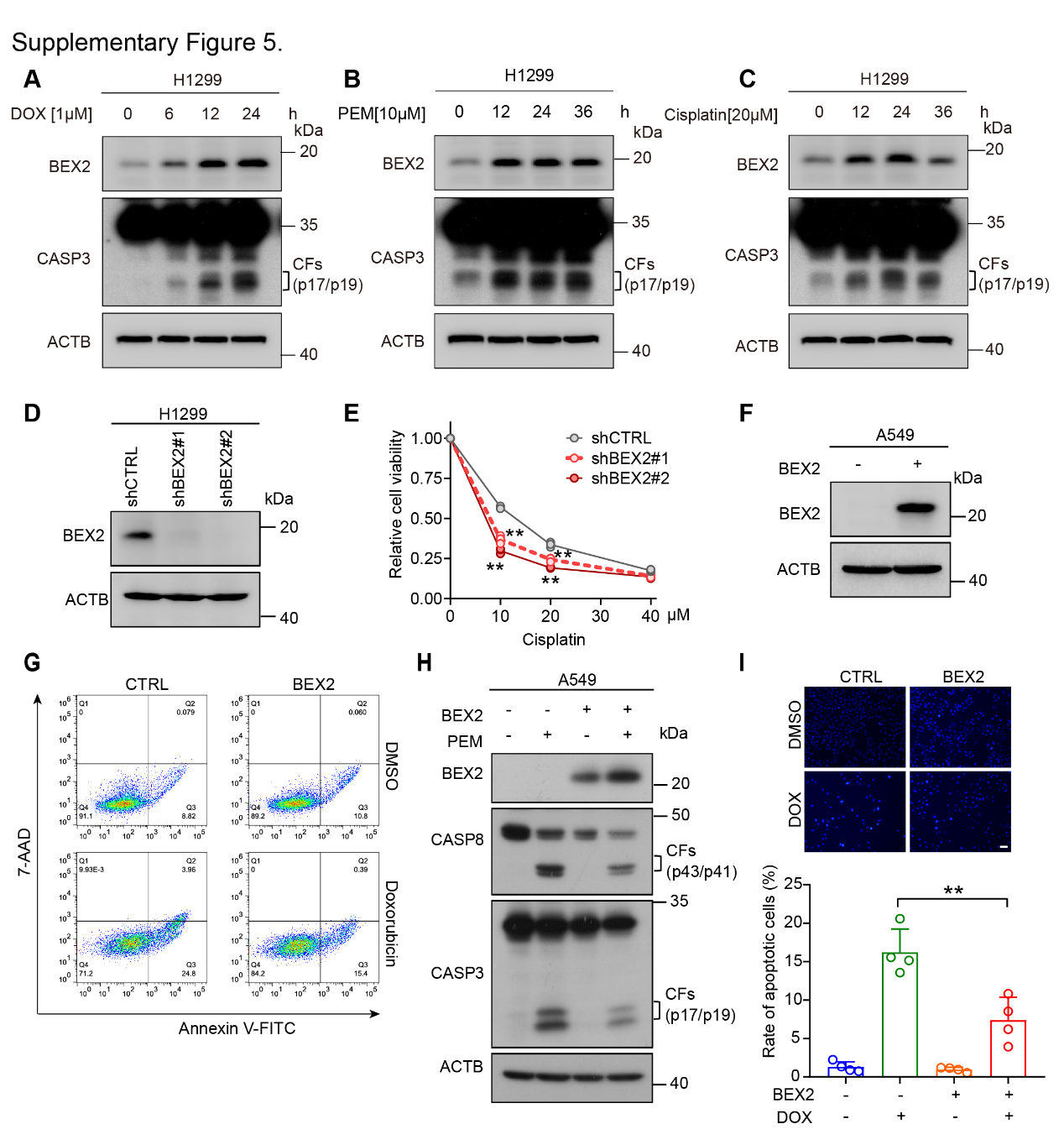


**Supplementary Figure 5. BEX2 inhibits chemotherapeutic agent-induced apoptosis.**

**(A-C)**. H1299 cells were incubated with doxorubicin (DOX 1μM) at different time points (0, 6, 12, 24 h) (A), pemetrexed (PEM 10μM) (B), and cisplatin (20μM) (C) at different time points (0, 12, 24, 36 h), and then cells lysates were analyzed by western blotting with the indicated antibodies.

**(D)**. Western blot analysis of stable knockdown of BEX2 in H1299 cells.

**(E)**. CCK-8 assays were performed in stably transduced H1299 cells, which were treated with cisplatin (0, 10, 20, or 40 μM) for 24 h. Data are presented as the mean±SD (*n* = 3), and statistical significance was assessed by two-way ANOVA. ***P*<0.01.

**(F)**. Western blot analysis of stable overexpression of BEX2 in A549 cells.

**(G)**. A549 cells were transfected with CTRL(pcDNA3.1) or BEX2 and then treated with doxorubicin for 24h, cells were stained by Annexin V-FITC/7-AAD and flow cytometry analysis.

**(H)**. A549 cells were transfected with CTRL(pcDNA3.1) or BEX2 and then incubated with pemetrexed for 36 h. Then, cell lysates were analyzed by western blotting with the indicated antibodies.

**(I)**. A549 cells were treated with doxorubicin for 24 h after overexpression of CTRL(pcDNA3.1) or BEX2. Hoechst 33342 staining analysis of cell apoptosis. Data are presented as the mean ± SD (*n*=3 independent experiments, 20 cells per experiment), and statistical significance was assessed by two-tailed Student’s *t*-test. ***P*<0.01. Scale bar: 50 μm.


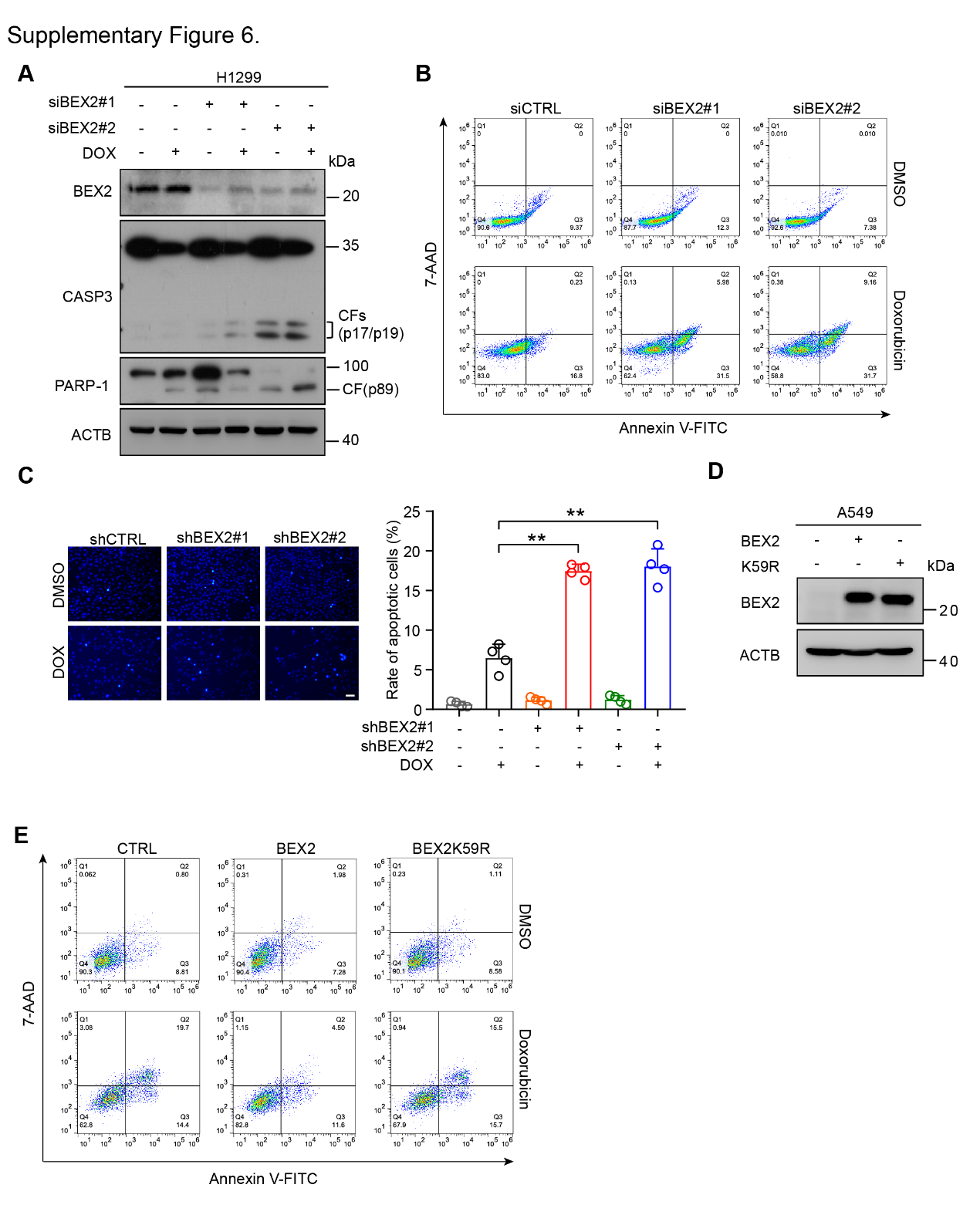


**Supplementary Figure 6. BEX2 inhibits chemotherapeutic agent-induced apoptosis.**

**(A)**. H1299 cells were transfected with siCTRL or siBEX2 and then treated with doxorubicin for 24h. Cell lysates were analyzed by western blotting with the indicated antibodies.

**(B)**. H1299 cells were transfected with siCTRL or siBEX2 and then treated with doxorubicin for 24h, cells were stained by Annexin V-FITC/7-AAD and flow cytometry analysis.

**(C)**. H1299 cells were treated with doxorubicin for 24 h after knockdown of BEX2. Hoechst 33342 staining analysis of cell apoptosis. Data are presented as the mean ± SD (*n*=3 independent experiments, 20 cells per experiment), and statistical significance was assessed by two-tailed Student’s *t*-test. ***P*<0.01. Scale bar: 50 μm.

**(D)**. Western blot analysis of stable overexpression of BEX2 and BEX2K59R in A549 cells.

**(E)**. A549 cells were transfected with CTRL(pcDNA3.1), BEX2 and BEX2K59R. After treatment with doxorubicin for 24h, cells were stained with Annexin V-FITC/7-AAD and detected by flow cytometry analysis.


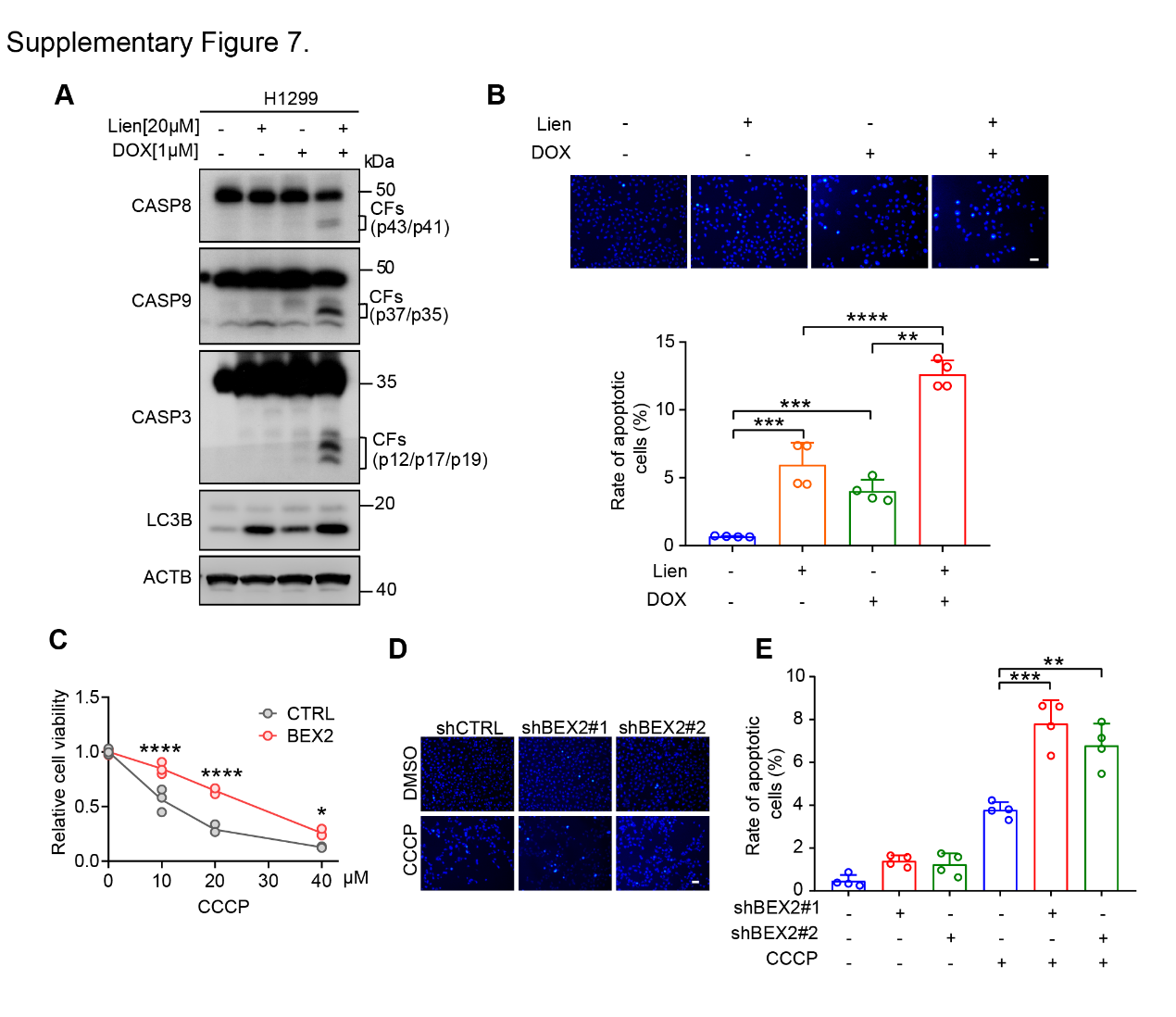


**Supplementary Figure 7. Inhibition of BEX2-regulated mitophagy sensitizes tumor cells to chemothrapy-induced apoptosis.**

**(A-B)**. H1299 cells were pretreated with liensinine (Lien, 20 μM) for 0.5 h and then combined with doxorubicin (DOX, 1 μM) for 24 h. Cell lysates were analyzed by western blotting with the indicated antibodies (A). Hoechst 33342 staining analysis of cell apoptosis. Data are presented as the mean ± SD (*n*=3 independent experiments, 20 cells per experiment), and statistical significance was assessed by two-tailed Student’s *t*-test. ***P*<0.01, ****P*<0.001, *****P*<0.0001. Scale bar: 50 µm (B).

**(C)**. CCK-8 assays were performed in stably transduced A549 cells, which were treated with CCCP (0, 10, 20, or 40 μM) for 24 h. Data are presented as the mean ± SD (*n*=3), and statistical significance was assessed by two-way ANOVA. *****P*<0.0001.

**(D-E)**. H1299 cells stably expressing shCTRL or shBEX2 were treated with CCCP for 24 h. Hoechst 33342 staining analysis of cell apoptosis. Data are presented as the mean ± SD (*n*=3 independent experiments, 20 cells per experiment), and statistical significance was assessed by two-tailed Student’s *t*-test. ***P*<0.01, ****P*<0.001. Scale bar: 50 μm.


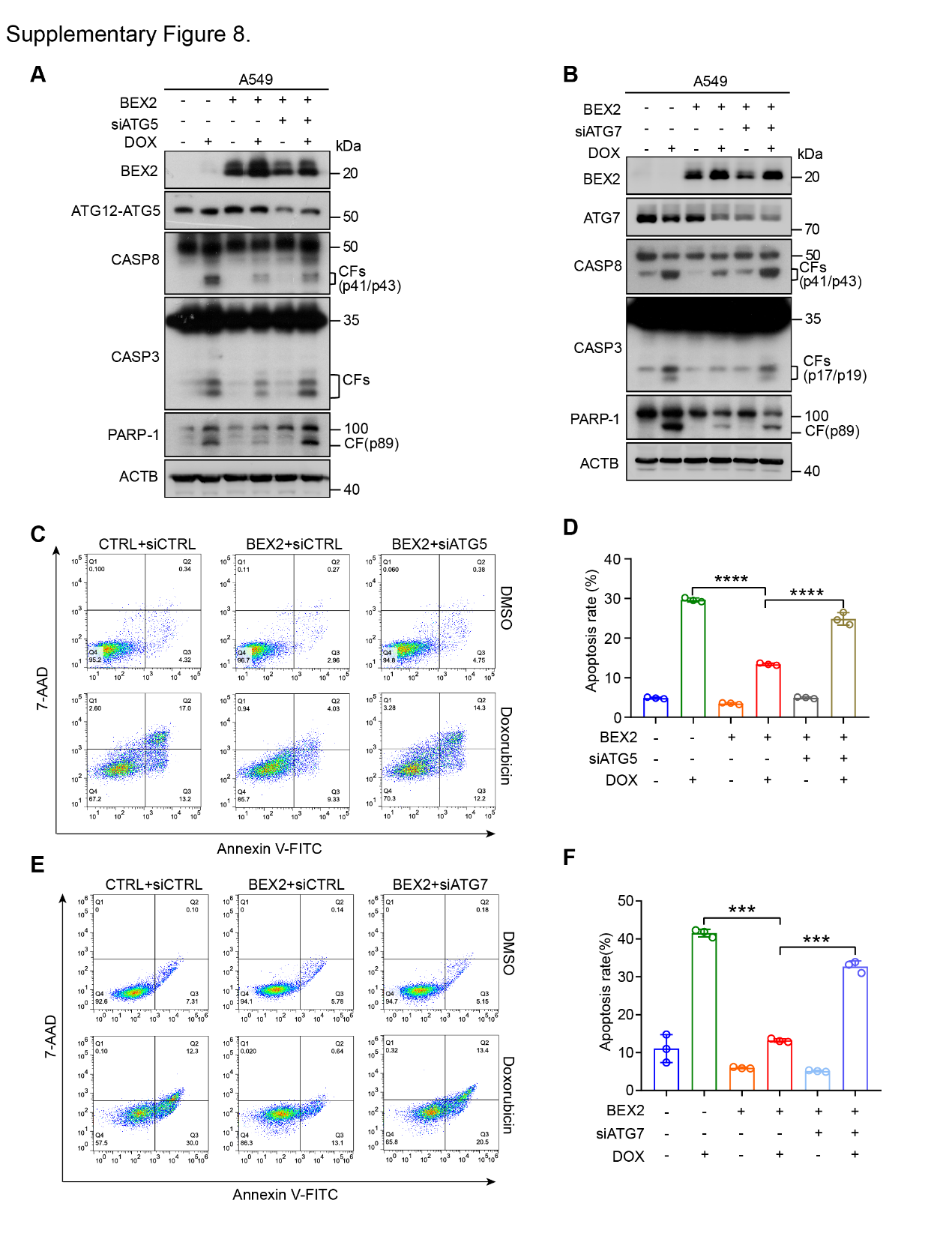


**Supplementary Figure 8. Inhibition of BEX2-regulated mitophagy sensitizes tumor cells to chemothrapy-induced apoptosis.**

**(A-B)**. A549 cells were transfected with BEX2 and ATG5 siRNA (A) or ATG7 siRNA (B), and then treated with doxorubicin for 24h. Cell lysates were analyzed by western blotting with the indicated antibodies.

**(C-F)**. A549 cells were transfected with BEX2 and ATG5 siRNA or ATG7 siRNA. After treatment with doxorubicin for 24h, cells were stained with Annexin V-FITC/7-AAD and detected by flow cytometry analysis. Data are presented as the mean ± SD (*n* =3), and statistical significance was assessed by two-tailed Student’s *t*-test. ****P*<0.001, *****P*<0.0001.
