# Supplementary figures and images for: Crotonylated BEX2 interacts with NDP52 and enhances mitophagy to modulate chemotherapeutic agent-induced apoptosis in non-small-cell lung cancer cells
Source: Cell Death Dis. 2023 Sep 30;14(9):645. doi: 10.1038/s41419-023-06164-6 (PMC10542755; doi:10.1038/s41419-023-06164-6)

**Original western blots**


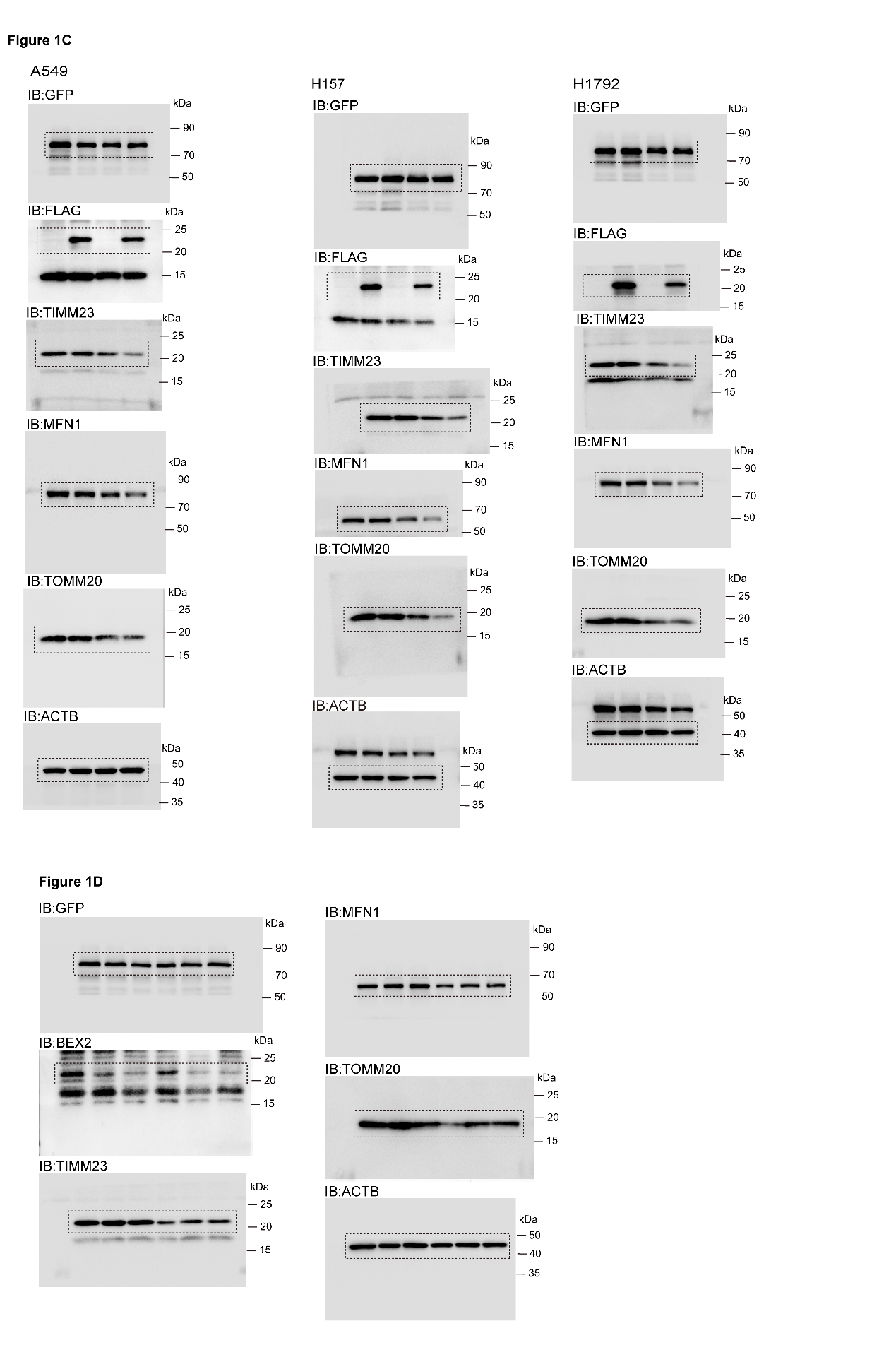


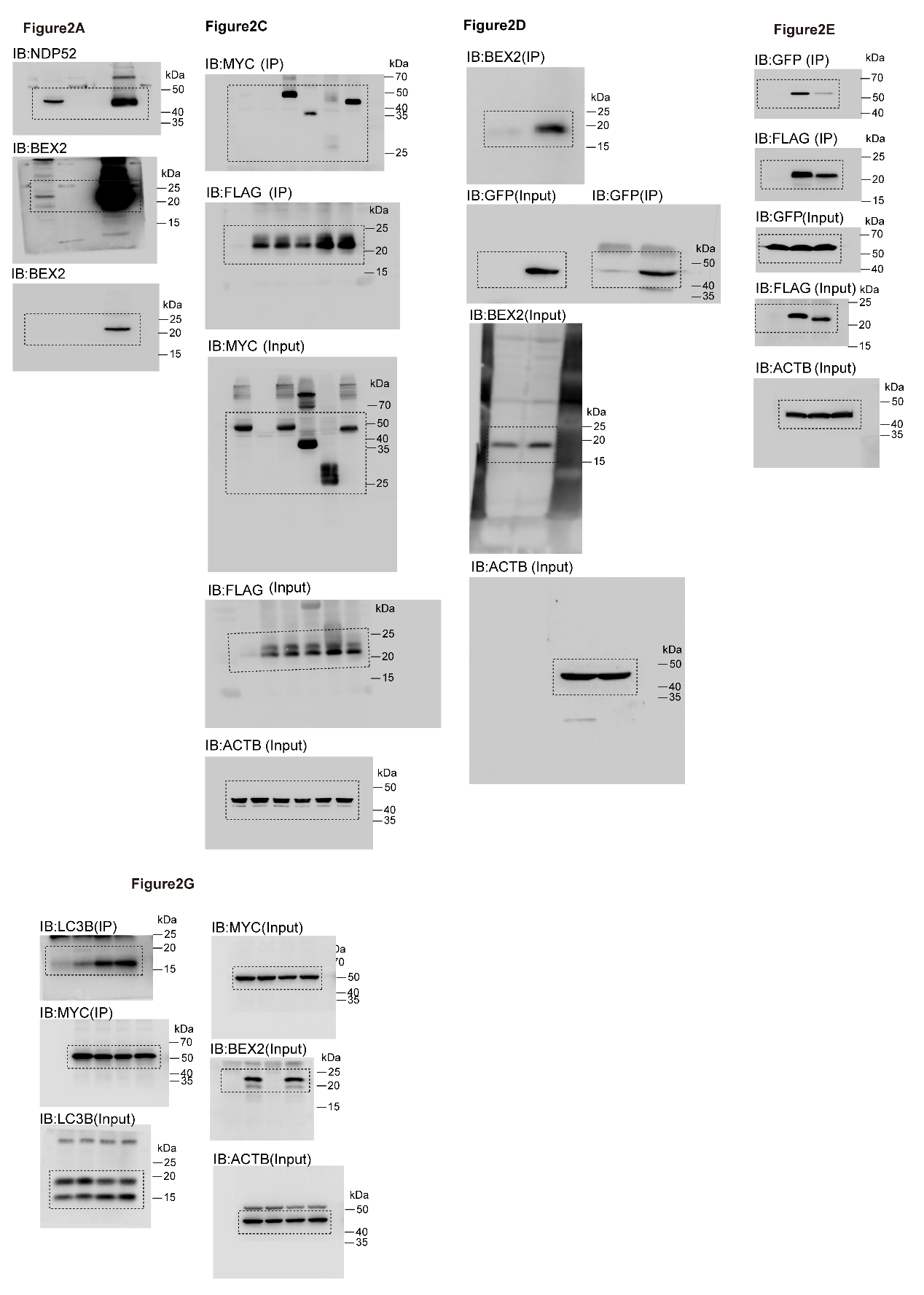


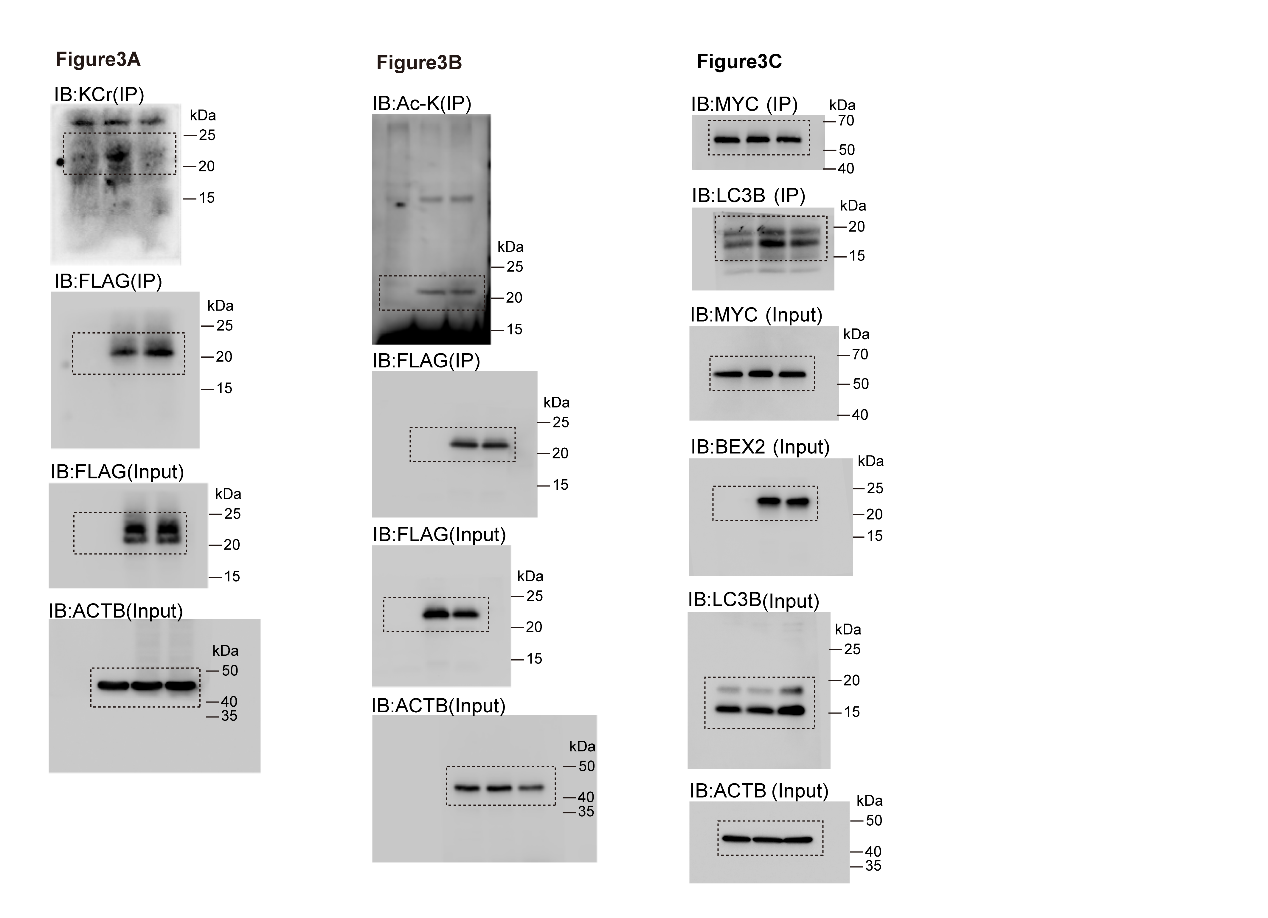


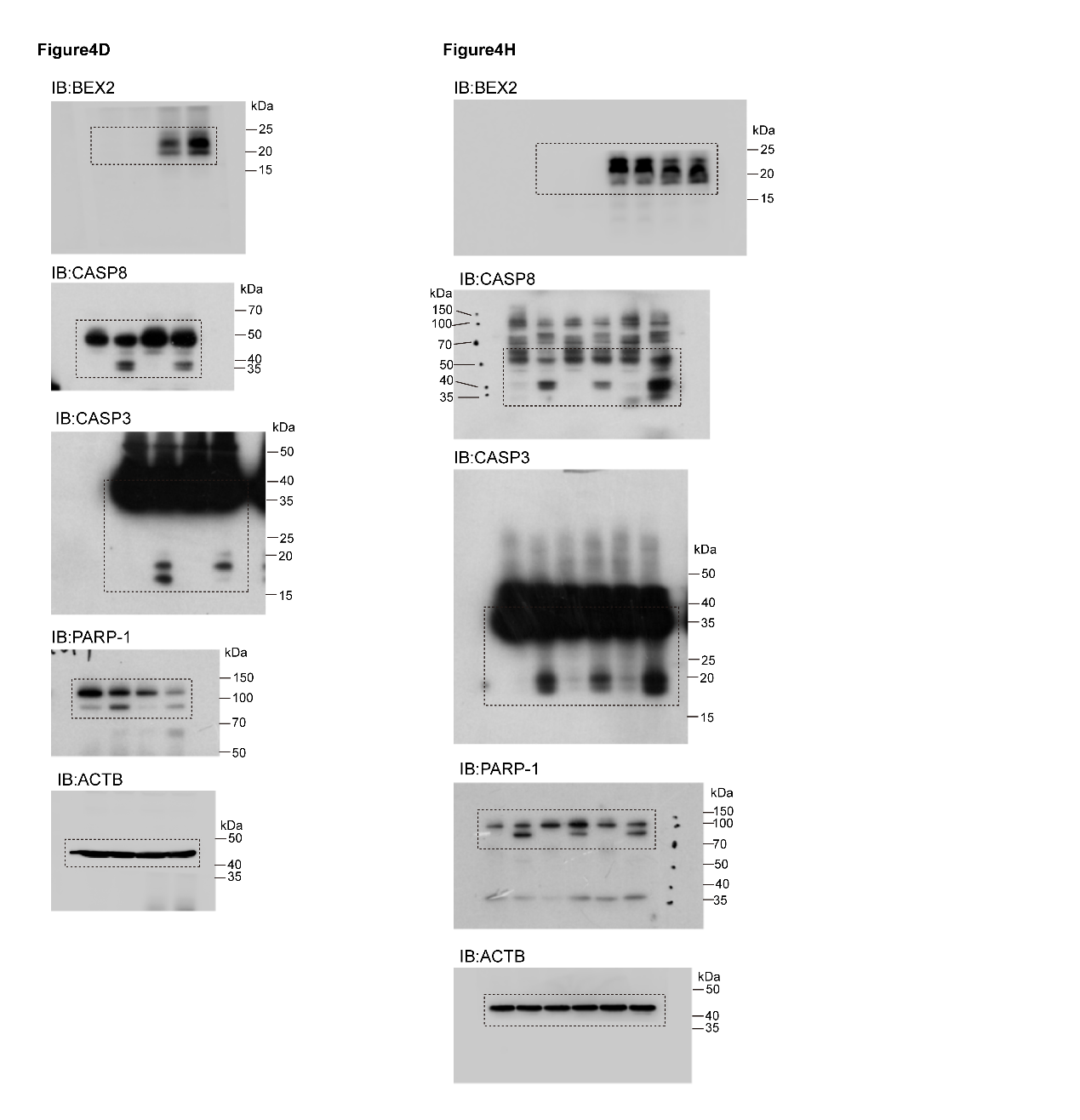


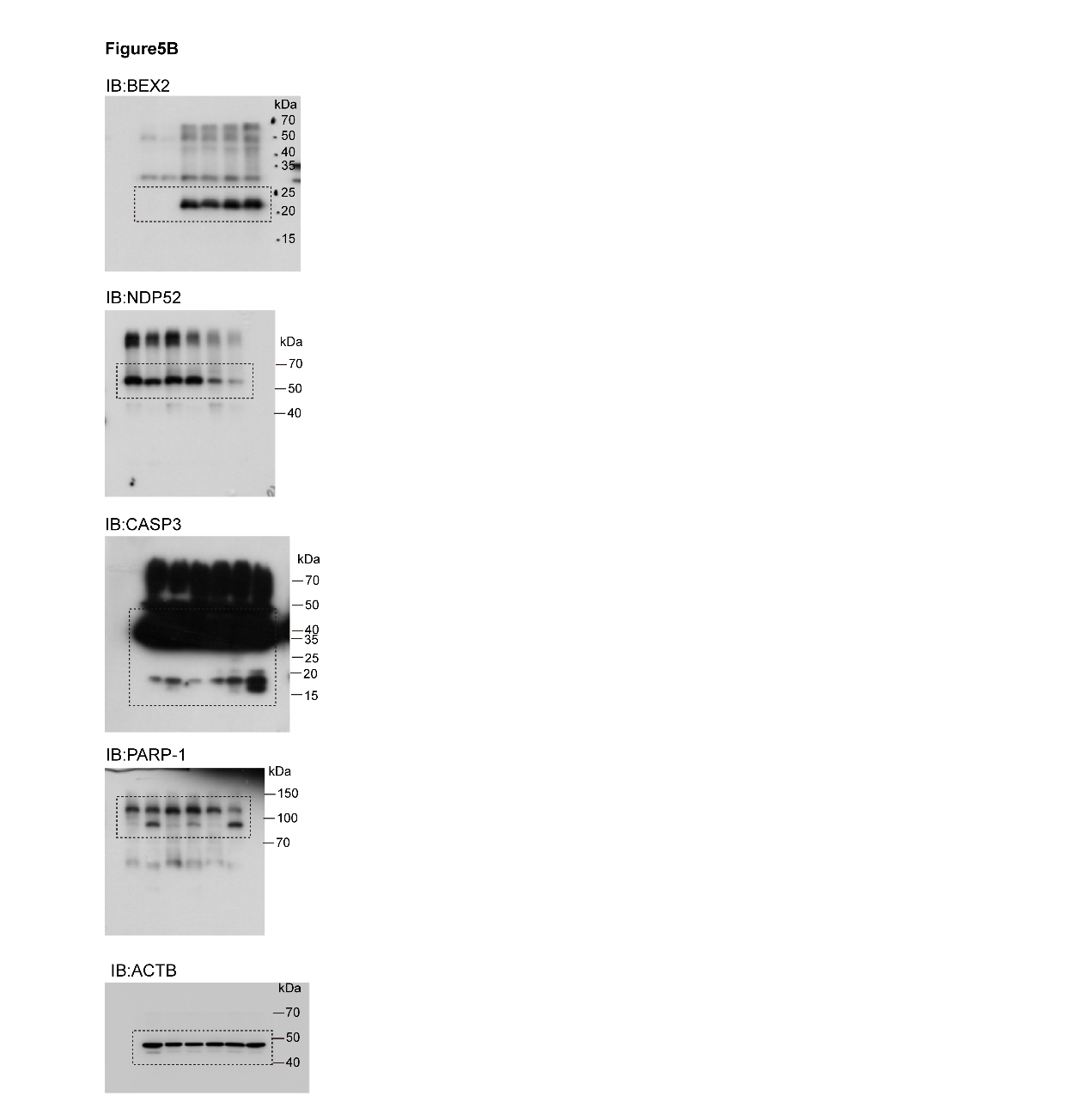


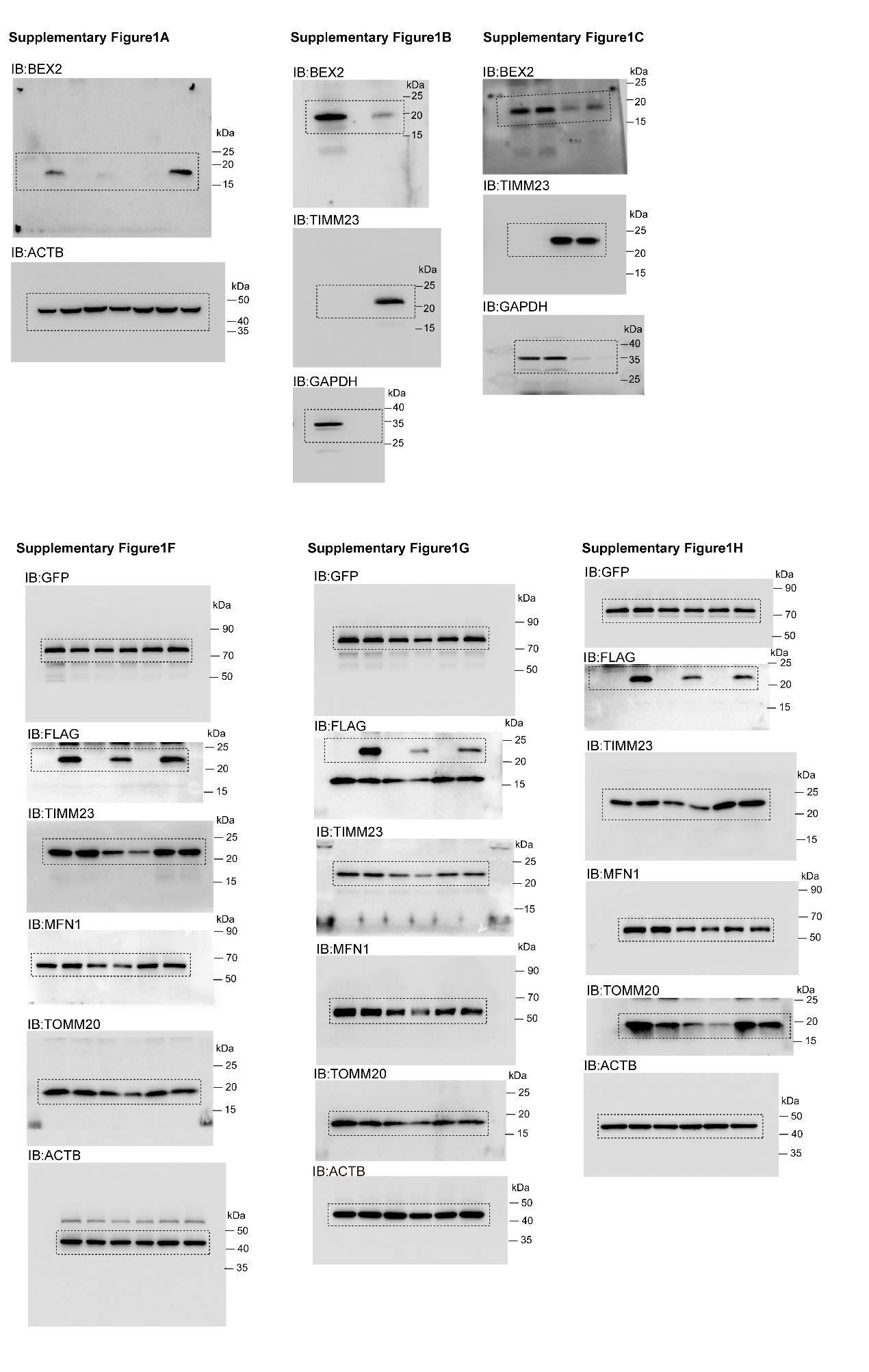


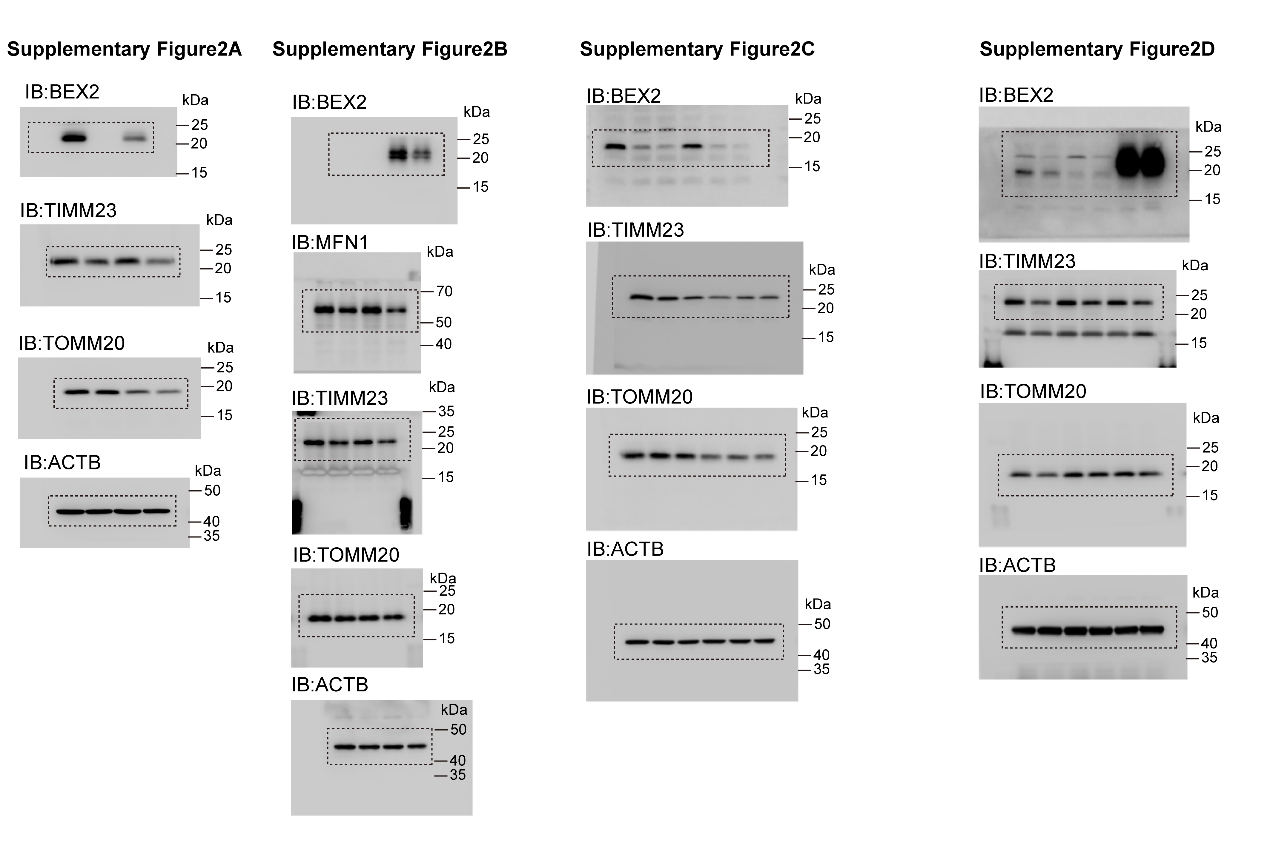


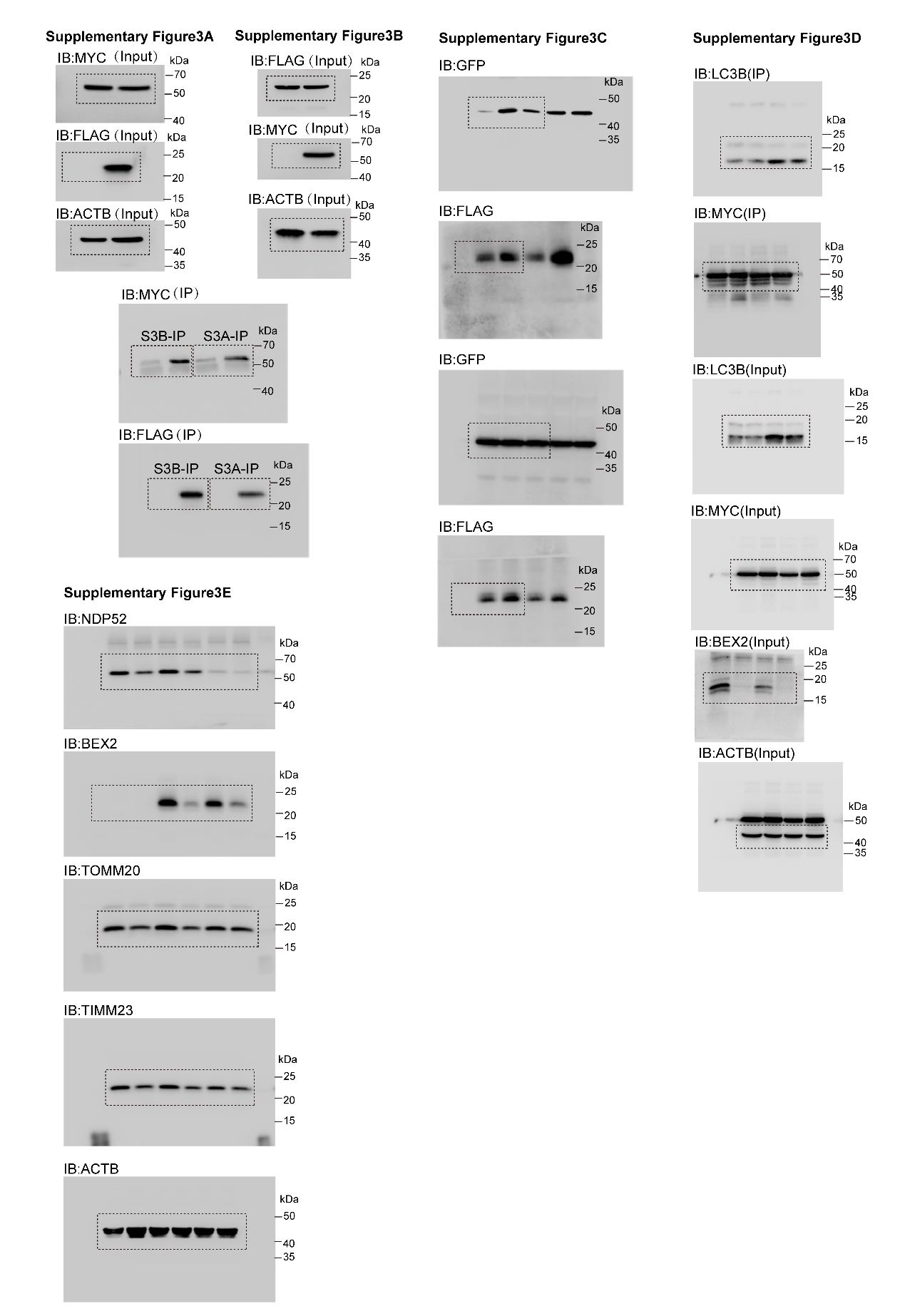


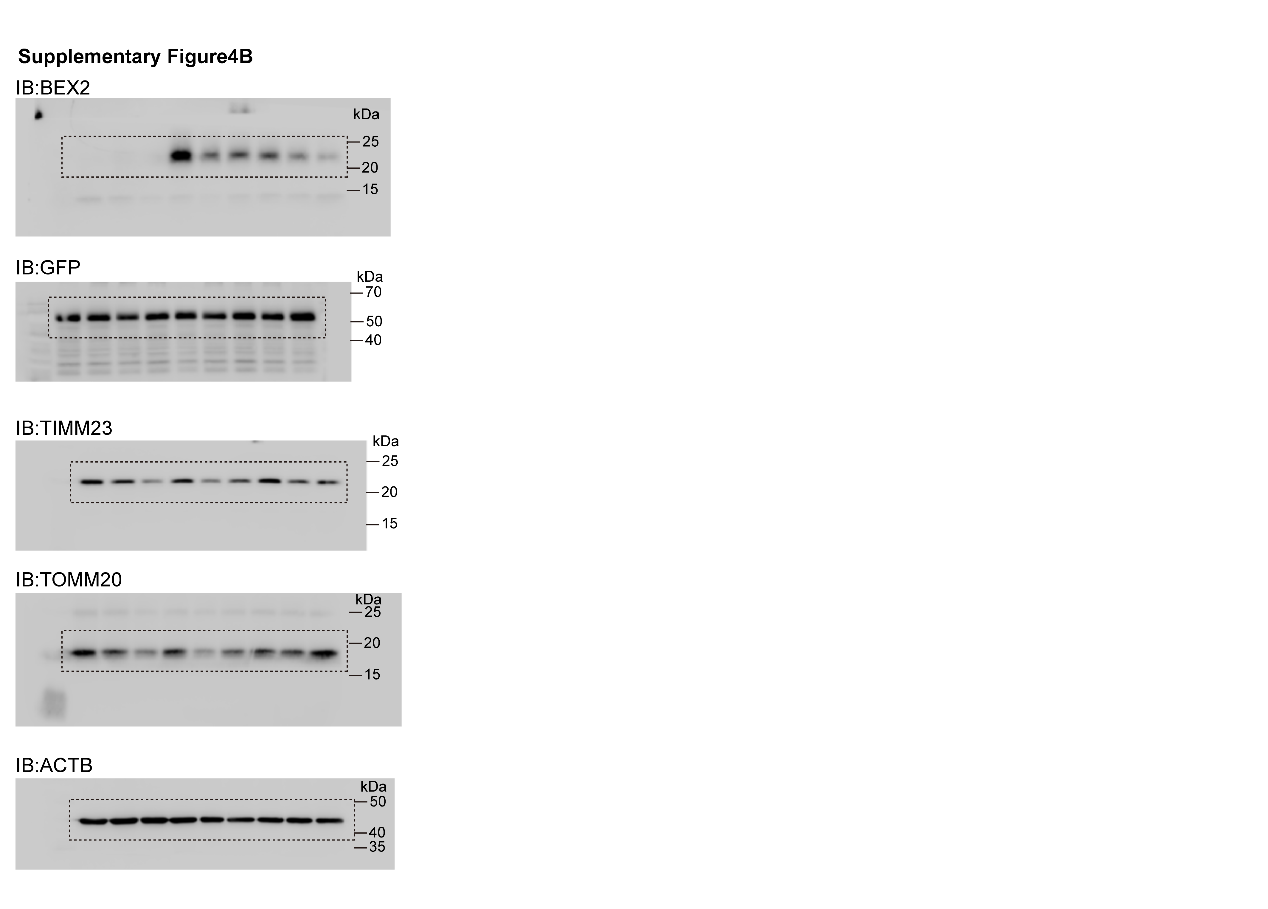


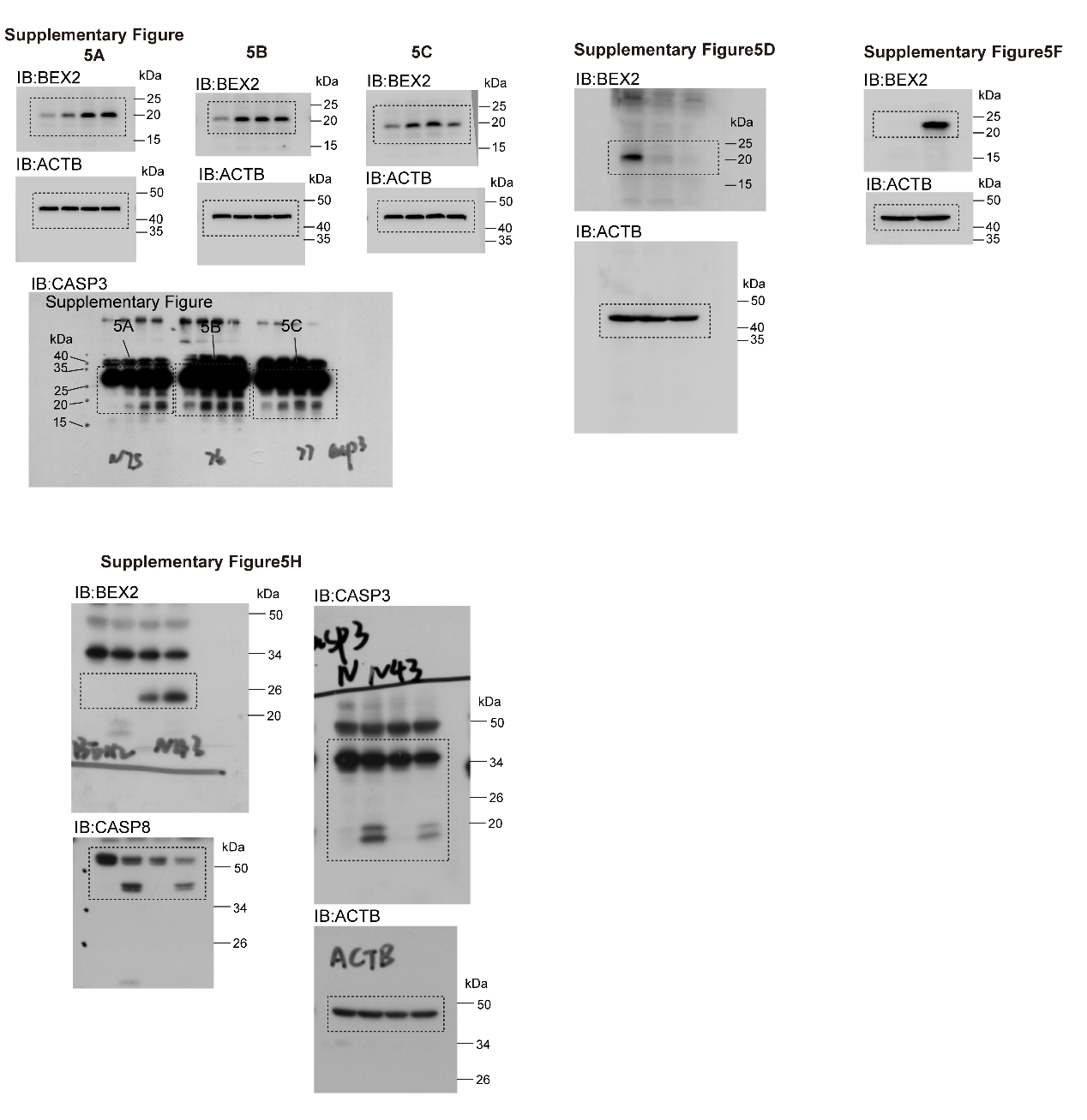


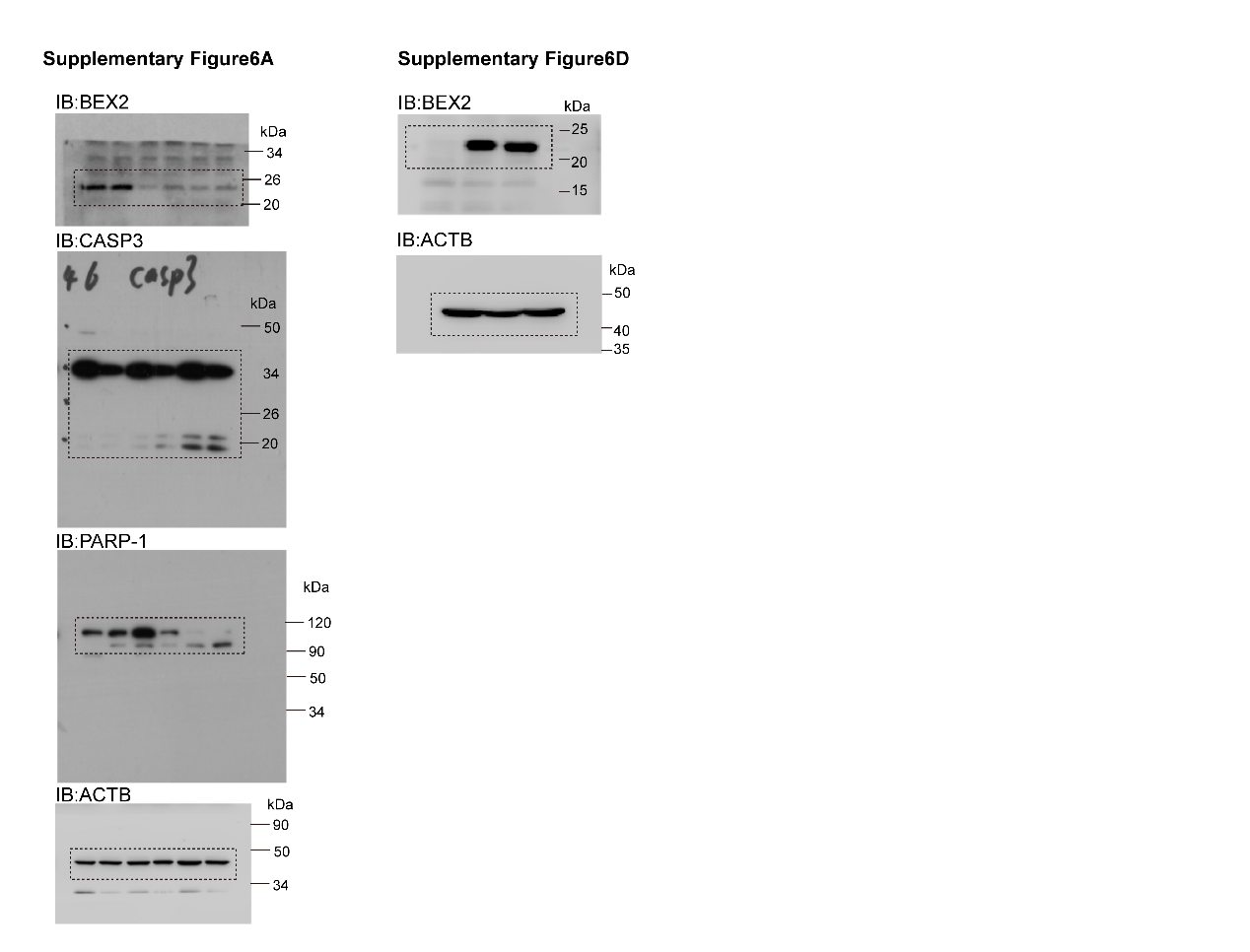


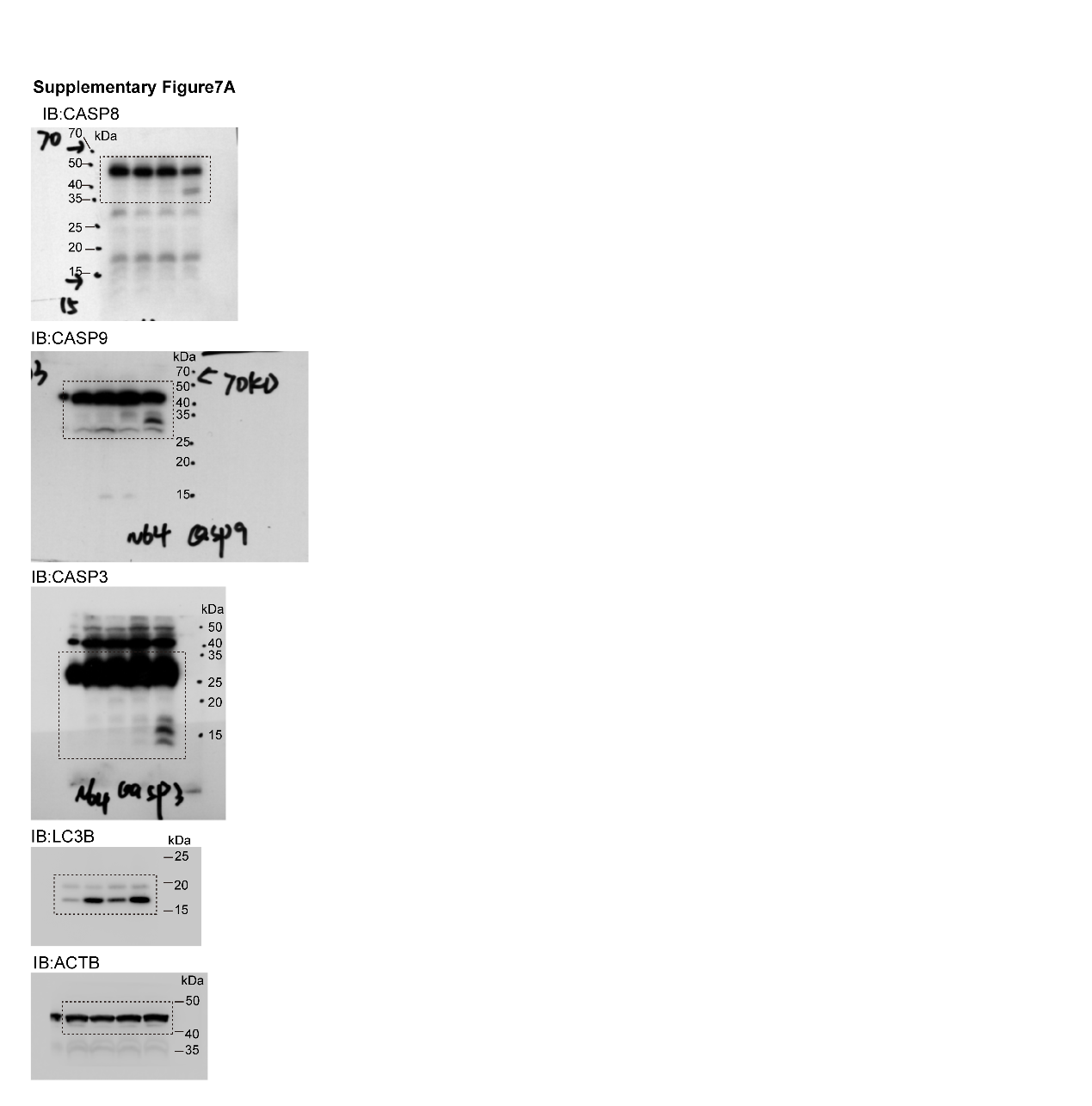


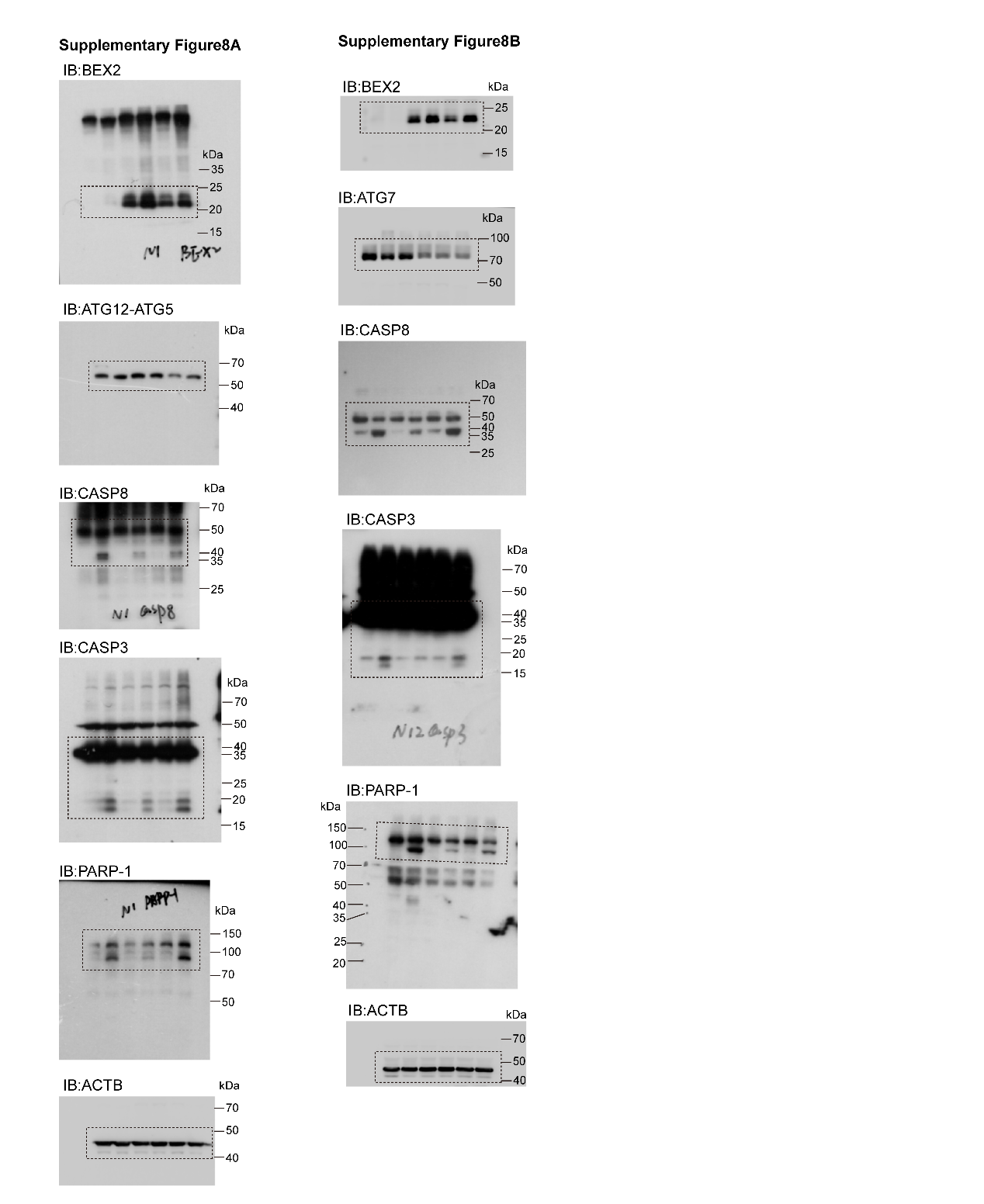

Supplement: Supplementary file 3 — Original western blots [file 41419_2023_6164_MOESM3_ESM.docx]
